# Sonochemical Synthesis of Low-Dimensional Nanostructures and Their Applications—A Review

**DOI:** 10.3390/ma17225488

**Published:** 2024-11-10

**Authors:** Grzegorz Matyszczak, Krzysztof Krawczyk, Albert Yedzikhanau, Konrad Głuc, Miłosz Szymajda, Aleksandra Sobiech, Zuzanna Gackowska

**Affiliations:** Department of Chemical Technology, Faculty of Chemistry, Warsaw University of Technology, Noakowski Str. 3, 00-664 Warsaw, Poland; krzysztof.krawczyk@pw.edu.pl (K.K.); albert.yedzikhanau.stud@pw.edu.pl (A.Y.); konrad.gluc.stud@pw.edu.pl (K.G.); milosz.szymajda.stud@pw.edu.pl (M.S.); aleksandra.sobiech.stud@pw.edu.pl (A.S.);

**Keywords:** sonochemistry, nanomaterials, nanostructures, quantum dots

## Abstract

Sonochemical synthesis is becoming a popular method of preparing various nanomaterials, including metals, carbons, oxides, and chalcogenides. This method is relatively cheap and responds to the challenges of green chemistry as it typically does not involve high temperatures, high pressures, inert atmospheres, or long reaction times in comparison to other conventional methods. The utilization of ultrasound in synthesis makes the elimination of toxic solvents possible, as well as the execution of the synthesis without the use of reducing and stabilizing agents, while receiving products with the same or even better properties. The application of ultrasound allows for the synthesis of various nanomaterials with different properties for use in fields such as catalysis, electrochemistry, medicine, and biosensors. The final product is influenced by multiple variables such as temperature, pH, reagents, capping agents, time of reaction, and the addition of dopants.

## 1. Introduction

### 1.1. Nanostructures

Within the last few decades, the scientific community has increasingly focused on so-called nanoscience. Despite concerns raised by mass media, nanomaterials (i.e., nanostructured materials) have been proven to be very useful in many applications as well as a driving force for nanoscience, which has led to more and more fascinating discoveries. The initially undefined phenomena thus had to be specified. Currently, the International Union of Pure and Applied Chemistry (IUPAC) provides a definition of nanoparticles as particles of different shapes, the size of which lies in the range of 10^−9^ to 10^−7^ m [1]. Due to their unique and extraordinary properties related to the phenomena in so-called mesospace (i.e., somewhere between molecule and bulk solid, however distinct from them), nanostructures have found applications in many disciplines. For example, nanomaterials are characterized by a relatively large surface-to-volume ratio, which is commonly in-demand in catalysis [2]. This fact, in addition to the high surface energy of nanomaterials, contributes to the ability of nanostructures to conduct various chemical reactions [2].

Nanostructures may be exemplified as, e.g., quantum dots (QDs) (which have great potential, e.g., in medicine for bioimaging [3,4]), nanometals (useful, e.g., in environmental protection), polymer nanomaterials, carbon nanostructures (that were utilized, e.g., for wastewater treatment [5,6]), and DNA or RNA nanospheres of extreme stability [7,8]).

The vast number of methods for the production of nanomaterials may be generally divided into two groups: “bottom–up” approaches, initially proposed by the father of nanotechnology—Richard Feynman, and “top–down” approaches [2]. The first approach builds nanostructures from individual atoms or molecules, while the latter is quite the opposite and utilizes processes of downsizing micro- and larger materials [2].

Another way to differentiate methods of nanomaterials synthesis is based on the nature of involved processes. Thus, we distinguish between physical and chemical methods of nanostructure preparation. Physical methods are, e.g., lithography, laser ablation, and molecular beam epitaxy [9]. Chemical methods include sol–gel reactions, ultraviolet and/or ultrasound-assisted syntheses, redox reactions, precipitation reactions, and those that involve microorganisms [2,9]. The main advantage of chemical over physical methods is better control of the size and shape of produced nanoparticles [3,10,11]. However, some of them require the usage of toxic reagents and high temperatures (e.g., the sol–gel method), which is a serious drawback in the context of large-scale production [12]. Nevertheless, the application of ultrasound in the synthesis of nanostructures may help overcome such problems.

### 1.2. Sonochemistry

**Figure 1:**
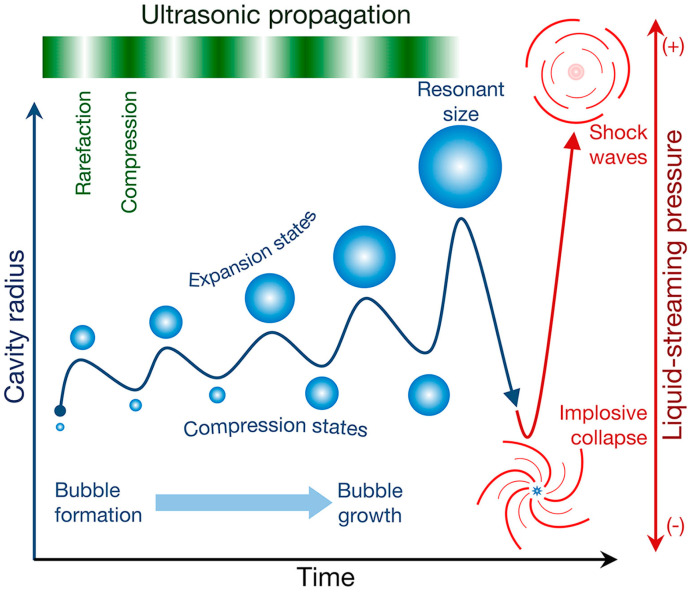
Schematic illustration of acoustic cavitation (reproduced from [14] under the Creative Common license).

The application of ultrasound for conducting chemical reactions is called sonochemistry. Thus, sonochemistry may be defined as a subdivision of chemistry that focuses on chemical changes caused by the interaction between ultrasound and the investigated system. The propagation of an ultrasound wave within a liquid medium may cause the decrease in solubility of gases within liquid due to pressure fluctuations. Under certain conditions, in the presence of crevices, a nucleation of bubbles may occur, which then grow, reach their maximum size, and suddenly collapse at the same time, releasing energy in the form of heat (a rise of temperature of order up to 10^4^ K), light (sonoluminescence) and pressure (order of 10^3^ atm), each of which may contribute to further chemical effects [5,13]. The schematic representation of the phenomenon of acoustic cavitation is presented in Figure 1.

Sonochemistry meets the requirements of “green chemistry” proposed by the United States Environmental Protection Agency. Briefly, they demand that the chemical processes should produce less waste while using fewer toxic reagents at the same time [14,15]. The application of ultrasound to chemical synthesis allows for the replacement of toxic, high-boiling solvents by less aggressive substances—even distilled or deionized water [5,13]. Moreover, it allows for the intensification of the process (i.e., its speeding up) without additional heating or catalyst. These are great advantages of sonochemistry over classical chemistry, which make it an interesting alternative [3,5,16].

## 2. Sonochemical Synthesis of Metal Nanostructures

Most of the sonochemical syntheses of metal nanostructures involve mixing a metal precursor with an appropriate solution containing other substances like reduction and stabilizing agents, and exposition of the reaction mixture to ultrasound. Final products are cleansed to obtain better purity and then dried.

In this summary, the sonochemical syntheses of nanostructures are divided into those that occur in bulk solution (Section 2.1.1) and those that happen on some surface (Section 2.1.2). Additionally, three other types of synthesis have been singled out, as they differ significantly in their course from the others. They are the synthesis of metal nanostructures using ultrafine bubbles (Section 2.1.3), a fabrication of metal nanostructures using the technique of ultrasonic nanoimprinting (Section 2.1.4), and an ultrasound-assisted method for phase structuring in metal alloys (Section 2.1.5).

**Figure 2:**
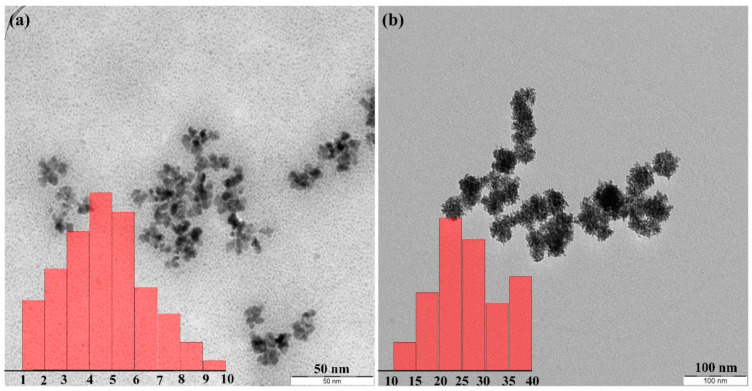
TEM image with the average size distribution of Pt NPs synthesized by (**a**) sonochemical method and (**b**) conventional method (reproduced with permission from [18]).

The advantages of sonochemical over conventional synthesis are their smaller particle size and control over the morphology of structures (Figure 2) by tuning operating parameters, higher colloidal stability, better crystalline properties, a smaller agglomeration of NPs, very good properties of the products, room-temperature synthesis, and fast and cost-effective processes [17,18,19].

### 2.1. Various Methods of Synthesis of Metal Nanoparticles

#### 2.1.1. Metal Nanoparticles Synthesized in the Solution

An analysis of the literature treating the sonochemical synthesis of metal nanoparticles reveals that in the solution (i.e., volume system), scientists have achieved the production of the following metals: gold (Au), platinum (Pt), silver (Ag), and copper (Cu). The summary of conditions of such syntheses is presented in Table 1. For the synthesis of Au NPs, typically HAuCl_4_ is used as a metal precursor. Au NPs were obtained in a variety of forms (like nanorods, icosahedral particles, nanosheets, and spherical particles) and sizes [4,17,20,21]. In a study performed by Yusof et. al., the shape and size of gold nanoparticles were influenced by the amount of formed radicals (reducing agents) and generated mechanical forces [20]. Other reducing agents for the synthesis of Au NPs were tryptophan (acting at the same time as a capping agent) and trisodium citrate [4,17]. Platinum nanoparticles were sonochemically obtained starting from two distinct metal precursors: K_2_PtCl_4_ and PtCl_4_ [18,19]. They were reduced by plant extract and NaBH_4_, respectively, forming ultrafine Pt NPs of approximate sizes in the range of 2–3 nm [18,19]. Silver and copper nanoparticles were sonochemically synthesized using their salts as precursors (AgNO_3_, Cu(CH_3_COO)_2_) [22,23,24]. In the case of the synthesis of Ag NPs, the addition of k-carrageenan (a stabilizer) was studied, and it was found that greater concentrations of stabilizer resulted in a higher yield of nanoparticles [22].

#### 2.1.2. Metal Nanoparticles Synthesized on the Surface

**Figure 3:**
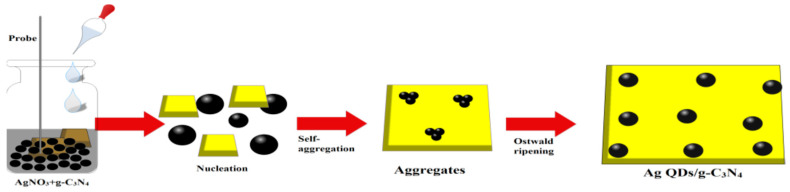
Mechanism of synthesis of Ag QDs on g-C_3_N_4_. Reproduced under the Creative Commons license from ref. [26].

Ultrasound-assisted surface processes may be utilized for the fabrication of metal–ceramic composites. Due to the shock waves and micro-jets created by sonication, the metal nanoparticles collide with the surface, and chemical bonds or weak interactions keep them there [25]. For example, silver QDs were synthesized in solution and in situ deposited on g-C_3_N_4_ [26]. The mechanism of such a process is presented in Figure 3. It involves mixing appropriate reagents (aqueous extract from ginseng root with g-C_3_N_4_ and adding a solution of AgNO_3_). As a result, the nucleation of silver QDs begins, and then the process of self-aggregation of QDs takes place [26]. Finally, through Ostwald ripening (a phenomenon during which small particles redeposit onto larger particles), the product is obtained.

**Figure 4:**
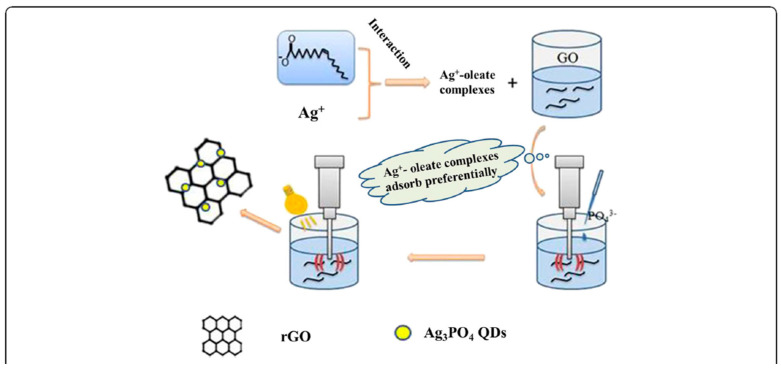
Schematic procedure of synthesis Ag_3_PO_4_ on graphite oxide. Reproduced under the Creative Commons license from ref. [27].

Similarly, a synthesis of Ag_3_PO_4_ QDs on reduced graphite oxide (rGO) was reported in ref. [27] and presented in Figure 4. Sodium oleate and AgNO_3_ were added to a graphite oxide solution (creating the Ag^+^-oleate complexes), and then Na_2_HPO_4_ aqueous solution was added drop by drop to the solution under ultrasonic irradiation. The obtained GO/Ag_3_PO_4_ QD composites were washed several times with hexyl alcohol and exposed to visible light irradiation and ultrasonic irradiation to obtain Ag_3_PO_4_ QDs on the reduced graphite oxide. In this case, similar to above (as in Figure 3), the QDs were uniformly dispersed on rGO.

Table 2 presents the overview of the basic parameters of selected syntheses of metal nanostructures that take place on a surface or some sort of support.

#### 2.1.3. Synthesis of Metal Nanostructures Using Ultrafine Bubbles (UFBs)

Ultrafine bubbles are smaller than 1 μm in diameter and have a very long lifetime in water, more than 2 months, as their upward velocity due to buoyancy is negligible [31]. UFBs can be generated by selecting the appropriate ultrasonic frequency: for example, using an ultrasonic cleaner at 42 kHz and an ultrasonic homogenizer at 20 kHz. Moreover, their generation rate increases with the increase in dissolved air content in pure water [32].

With the UFBs, synthesis can be carried out without reducing and capping agents as in the case of fabrication of size-controlled Au@Pd core–shell NPs by ultrasound and UFBs [31]. Moreover, in this research, the concentration of UFBs was changed, and a relationship was found between the mean diameter of the bimetallic core–shell NPs and ultrafine bubble concentration [31]. As the concentration of UFBs became higher, the size of NPs became smaller. Without UFBs, the mean particle diameter was 30 nm, and with the high concentration of UFBs, it can decrease to 6 nm [31]. The explanation for decreasing the size of NPs in this method is that by ultrasound, UFBs become nuclei of cavitation, so the amount of cavitation and the amount of reducing species increases. The process of nucleation prevails in the solution.

**Figure 5:**
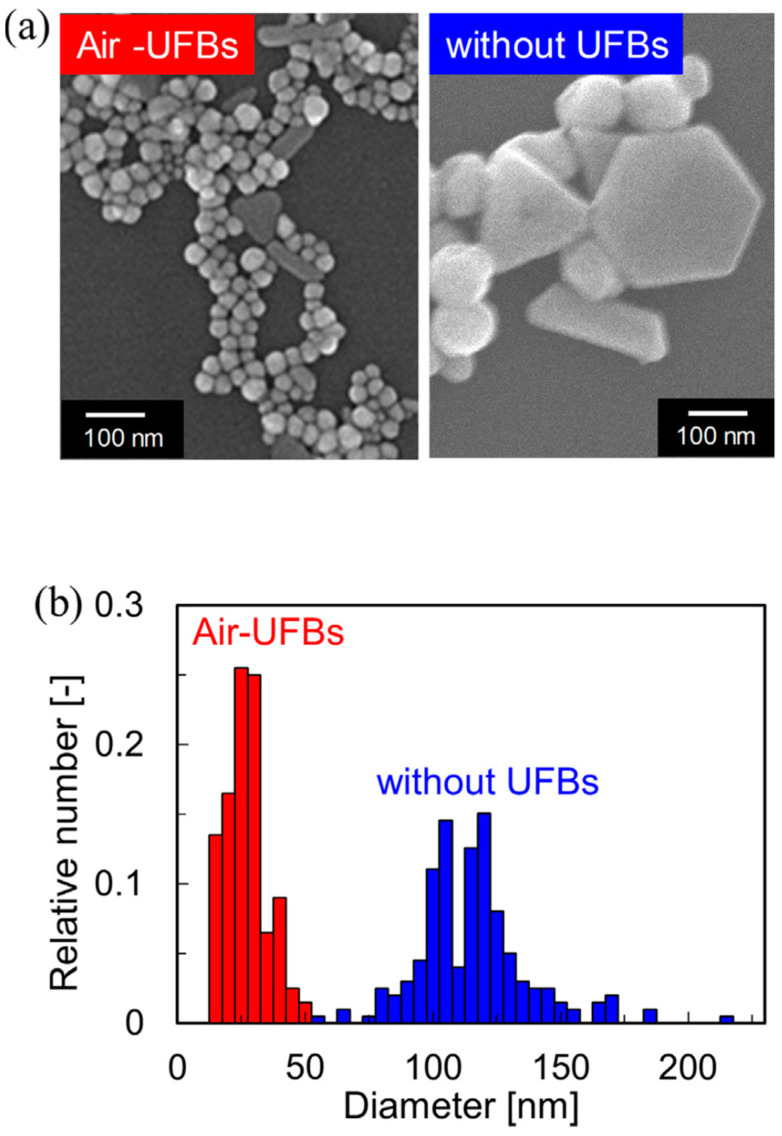
(**a**) The difference in appearance of the synthesized gold NPs; (**b**) the difference in size of the synthesized gold NPs. Reproduced with consent from ref. [33].

The same conclusions came out from the sonochemical synthesis of Au NPs with UFBs (results presented in Figure 5) [33]. Nanoparticles synthesized with UFBs are smaller and more spherical. Additionally, the Au NPs were very stable in a solution, because Au NPs were electrostatically adsorbed onto UFBs [33].

#### 2.1.4. Synthesis with the Use of the Ultrasonic Nanoimprinting Technique

**Figure 6:**
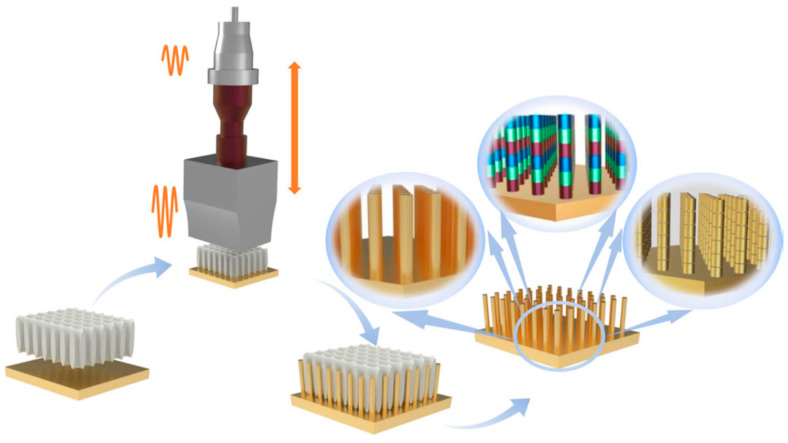
Schematic of ultrasonic nanoimprinting: anodic aluminum oxide used as mold is placed on the substrate–metal foil, next there is the ultrasonics nanoimprinting process, and then the look of products: nanowires, one-dimensional heterojunctions and nanowires with multiple nanogaps. Reproduced under the Creative Commons license from ref. [34].

The ultrasonic nanoimprinting technique reported by Ge et al. uses ultrasonic strikes (20 kHz) with concentrated energy to shape the substrate surface into nanostructures (see Figure 6) [34]. The controlled process gives opportunities for creating complex multi-compositional nanostructures made of virtually all solid materials. Using this technique, Au nanowires and heterojunction nanowires like Au–Bi, and Au–Sn (even if the mechanical properties of these metals are at the end of the spectrum) were fabricated [34].

#### 2.1.5. Ultrasound-Assisted Structuring of the Phase in Metal Alloys

**Figure 7:**
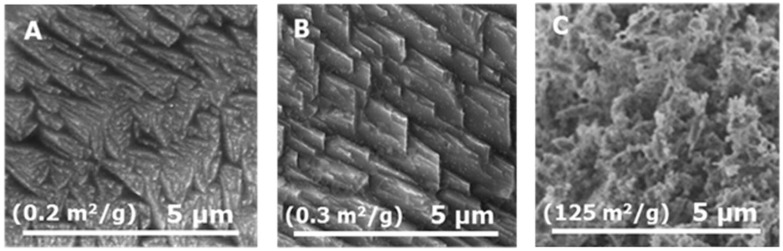
SEM images and surface areas of the initial (**A**), high-intensity ultrasound treated in ethanol (**B**) and water (**C**) AlNi (50 wt. % Ni, 50 wt. % Al) alloy particles. Reproduced under the Creative Commons license from ref. [35].

Another interesting technique involving ultrasound is reported in ref. [35]. Structuring of the phase occurs due to interfacial redox reactions and solid-state phase transformations using temperature. High-intensity ultrasound is a tool to change the surface composition and morphology of metals. This research studied the changes caused by sonication in different solvents (ethanol, ethylene glycol, decane, and water) for 60 min operated at 20 kHz (see Figure 7).

### 2.2. Relationships Between Parameters of Synthesis and Features of Obtained Nanostructures

There are many different parameters of sonochemical synthesis such as ultrasound power, frequency, sonication time, atmosphere above reaction mixture, dissolved gas, selection of solvent and each reagent (reducing, stabilizing, capping agents), and concentration of reagents. Each of these parameters has some impact on the synthesis.

Furthermore, there are many different features of nanostructures like size, shape, aggregation of NPs, purity, stability, and other special properties.

**Figure 8:**
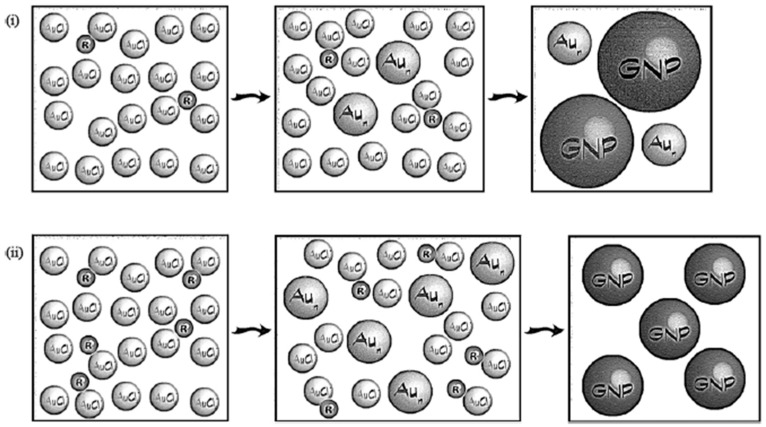
The effect of the concentration of radicals in the solution is shown by the example of gold nanoparticle (GNP) synthesis. (**i**) Dominance of the growing process when there are few radicals and (**ii**) dominance of the nucleation process when there are a lot of radicals. Reproduced with consent from ref. [20].

The size and shape of metal nanoparticles are mostly determined by two competing processes in a solution: nucleation (metal ion reduction in bulk) and growth (metal ion reduction on nuclei). Which one prevails depends on different factors, for example, the concentration of radicals in the solution, which depends on more different synthesis conditions like the power of ultrasound, solvent, reduction agent, etc. The relationship between the radical concentration and dominance of each process is presented in Figure 8.

#### 2.2.1. Effect of the Ultrasound Power

**Figure 9:**
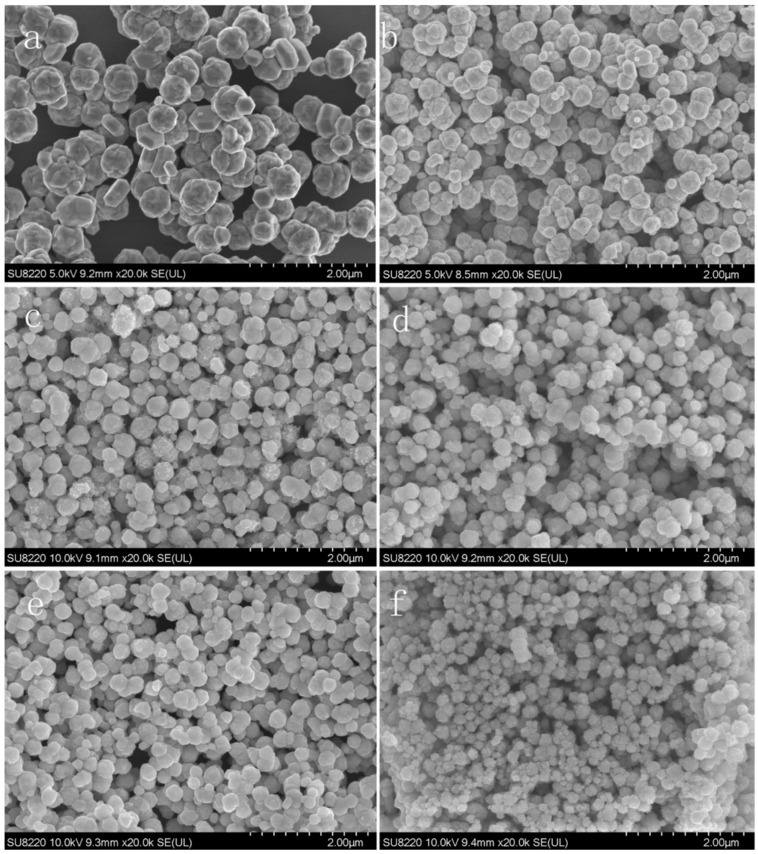
SEM morphologies of the as-synthesized Cu NPs under different ultrasonic powers. As the ultrasonic power increases from 0 (**a**) to 500 W (**f**), the average diameter of the Cu nanoparticles decreases from 520 to 167 nm. (**a**) 0 W, (**b**) 80 W, (**c**) 120 W, (**d**) 160 W, (**e**) 200 W, (**f**) 500 W. Reproduced under the Creative Commons license from ref. [24].

There is a correlation between the power of ultrasound and the size of metal nanoparticles produced in the sonochemical synthesis. Higher acoustic power levels generate a higher number of cavitation bubbles, and therefore, a higher number of radicals, so the process of nucleation prevails, and the size of NPs decreases [4,20,24]. An example of such a relationship is presented in SEM images of synthesized Cu NPs in Figure 9.

#### 2.2.2. Effect of the Ultrasound Frequency

Hansen et al. investigated the synthesis of Pt NPs and compared the obtained results for sonication under 20 and 408 kHz (bringing these two different systems to similar parameters such as reduction rate and NPs sizes) and concluded that the sonochemical synthesis at 408 kHz was more efficient and yielded nanoparticles of higher purity [19].

#### 2.2.3. Effect of the Concentrations of Reagents

**Figure 10:**
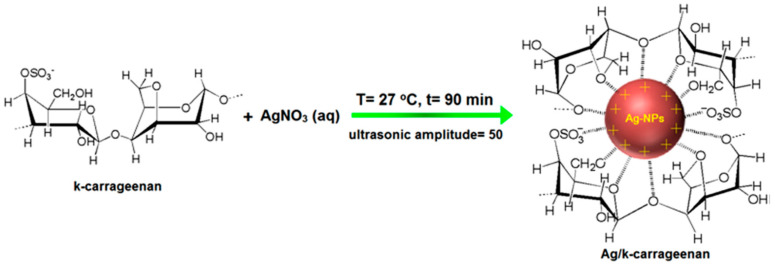
The stabilizing and capping effect is illustrated in the example of k-carrageen and Ag NPs. Reproduced under the Creative Commons license from ref. [22].

In the synthesis of silver NPs with κ-carrageenan as a stabilizer, and radicals generated due to ultrasonic irradiation (as a reducing agent), the concentration of the stabilizer was altered [22]. It was found that when the concentration of κ-carrageenan increased, the number of Ag-NPs also increased because of the increasing number of radicals concentration in the solution (radicals from water sonolysis reacted with stabilizer). In the solution, also the interaction between the Ag-NPs charged groups and the κ-carrageenan takes place (capping effect, see Figure 10).

#### 2.2.4. Effect of Dissolved Gases on the Sonochemical Synthesis

The dissolved gas in aqueous solutions plays a critical role in sonochemical reactions. In ref. [21], the authors checked the influence of argon, nitrogen, oxygen, and hydrogen as purged gases on the gold NPs synthesis. It turned out that the formation of smaller spherical AuNPs in the H_2_-purged solution compared with other gases would be attributed to the increase in the number of hydrogen radicals due to the reaction of hydrogen with OH radicals generated from the sonolysis of water. While oxygen in solution can interact with hydrogen radicals to produce water molecules and larger Au NPs, nitrogen and argon do not react with hydrogen radicals and do not affect the size and shape of Au NPs [21].

Furthermore, it is also important to have a constant appropriate gas atmosphere inside the flask to prevent any oxidation of the nanostructure surface during the synthesis [23].

#### 2.2.5. Effect of Solvent in the Sonochemical Synthesis

A very important parameter of the synthesis is the selection of solvent. Its key features are viscosity, surface tension, and temperature [30]. In the research conducted by Okoli et al., various solvents were compared: hexadecane, ethanol, ethylene glycol, polyethylene glycol, and selected ionic liquids [30]. The results show that the smallest nanostructures were achieved on carbon materials functionalized with ionic liquids in ethanol/deionized water mixture solvent rather than in any organic solvent [30].

Other significant changes can be made by synthesizing metal nanostructures in an emulsion system instead of a classical single phase [36]. The research shows that synthesizing QDs in emulsion is much faster because the heterogeneous nucleation has a lower energy barrier and leads to more frequent and more effective cavitation events to drive the reactions [36].

The capping effect of tryptophan was also described by Baral and co-authors and relies on π–metal interactions between gold nuclei and absorbed tryptophan, which is dimerized by ultrasound [17]. This determines the gold nuclei to grow as 2D nanosheets [17].

#### 2.2.6. Stability of Sonochemically Synthesized Metal Nanostructures

It has been described that the spherical Au NPs fabricated through sonochemical synthesis can remain unchanged for more than one month [21]. This is because chloride ions (originating from the metal precursor, HAuCl_4_) adsorb on the Au NPs surface. This fact was also confirmed by the addition of HCl and NaCl and the observation that Cl^−^ ions, because of adsorption, also affect crystal growth (plate-like particles were additionally present in the product) [21].

It was also demonstrated that metal NPs can achieve good long-term stability—Ag NPs do not change their optical properties even over 6 months [23]. However, a short exposure of Ag NPs to UVA radiation caused a small increase in the NPs concentration and improved their optical properties versus some decrease in concentration when exposing Ag NPs to UVC radiation [23].

### 2.3. Applications of Sonochemically Synthesized Metal Nanostructures

Sonochemically synthesized metal nanostructures have found various applications presenting catalytic and antimicrobial activities (see Table 3). In the field of catalysis, sonochemically derived gold nanosheets and Au@Pd core–shell nanoparticles were used in the reduction of 4-nitrophenol to 4-aminophenol [17,31]. Both prepared nanostructures showed relatively high catalytic activity. The sonochemically obtained gold nanosheets exhibited also excellent recyclability, while Au@Pd core–shell NPs were possible to obtain without the usage of capping or reducing agents, which contributed to the uncapped surface and consequently to the superior catalytic performance [17,31]. High-intensity ultrasound allowed the activation of initially inactive AlNi alloys to produce hydrogen due to the ultrasonically induced temperature gradient that led to changes in their surfaces [35].

**Figure 11:**
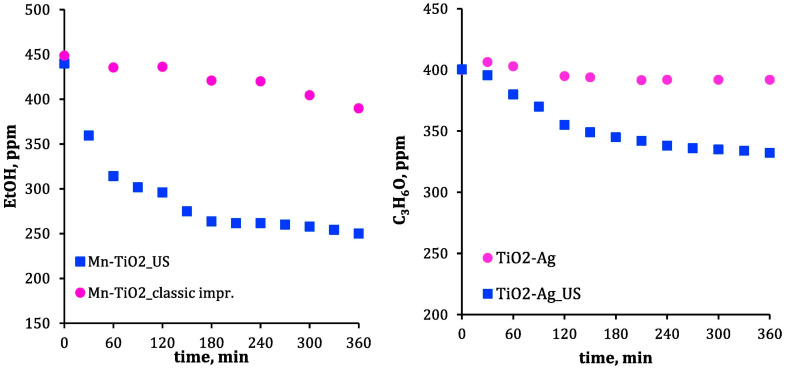
Change in concentration of ethanol (**left**) or acetone (**right**) during visible light irradiation for TiO_2_ photocatalyst coated with different metal NPs. Blue squares indicate points for catalysts prepared by ultrasound-assisted impregnation, while the pink circles indicate points corresponding to catalysts prepared using classical impregnation. Reproduced from ref. [29] under the Creative Commons license.

Photocatalytic applications of sonochemically derived metal nanostructures focused mainly on the degradation of organic compounds such as dyes and organic pollutants of water [29]. As an example, for this purpose, heterostructured nanoparticles of composition Ag/Cu/Mn on TiO_2_ (Ag-TiO_2_, Cu-TiO_2_, Mn-TiO_2_) were used [29]. It turned out that bare TiO_2_ was a far worse photocatalyst than the modified TiO_2_, while the best photocatalytic activity was exhibited by Mn-TiO_2_ NPs [29]. Interestingly, Ag-TiO_2_ and Mn-TiO_2_ prepared by the ultrasound-assisted impregnation showed better catalytic activity than in the case of conventional impregnation in the removal of acetone and ethanol, respectively (see Figure 11) [29]. Another example of a photocatalytic process utilizing metal NPs is hydrogen evolution on Ag QDs supported by g-C_3_N_4_ [26]. The combination of Ag with g-C_3_N_4_ enhances the separation of photogenerated electron–hole pairs [26]. This nanomaterial presented also an antimicrobial activity comparable to the commercial antibiotic chloramphenicol [26].

In the field of electrocatalysis, Pd_3_Mo NPs supported by carbon substrates were used for the oxygen reduction reaction which is important for fuel cell applications [30]. Sonochemically obtained nanocomposites of Pt–Carbon black were also utilized in the field of electrocatalysis [6,37].

## 3. Sonochemical Synthesis of Carbon Nanostructures

Carbon nanostructures such as carbon nanotubes, fullerenes, nanofibers, nanodiamonds, graphene and its derivatives, and carbon quantum dots (CQDs) are materials sought after for their unique and useful properties in many different fields such as electronics, optics, medicine, or catalysis [38,39,40]. Some of the features that these kinds of nanomaterials are known to exhibit include high fluorescence and photoluminescence [39,41,42], high electrochemical performance [43], high electrical conductivity, high storage performance [44], and high biocompatibility (low toxicity) [40,45].

To synthesize such materials, a variety of methods can be used, which can be divided into “top–down” and “bottom–up” approaches depending on the source of carbon used for synthesis [46]. Employing some of these methods can entail the usage of strong chemicals or complicated, energy-consuming instruments to process bulk sources of carbon. However, with the help of ultrasound techniques, it is possible to acquire desired products in milder conditions and with a significantly smaller costs even when there is a need to process the source of carbon used before it can be used for proper sonochemical synthesis [44,47,48,49].

### 3.1. Equipment Used for Sonochemical Synthesis of Carbon Nanostructures

**Figure 12:**
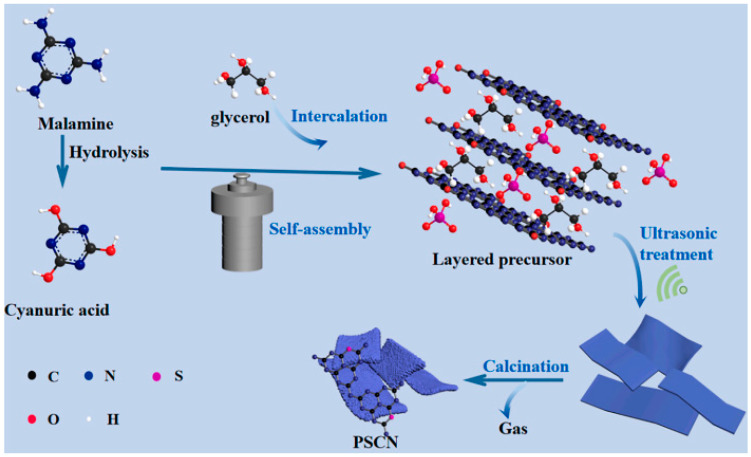
Schematic diagram on the fabrication of PSCN photocatalyst by combining ultrasonic treatment with other methods [51].

Standard experimental setups for sonochemical synthesis of carbon nanomaterials consist of a source of ultrasonic irradiation such as ultrasonic cleaners [46,48] or ultrasonic probes [40] and equipment used for purification such as centrifuges, filters, and drying ovens. Depending on the type of carbon nanostructures that are synthesized or materials used for synthesis, researchers use different types of additional equipment besides standard ones. An example of that can be found in the synthesis of graphite oxide and graphite QDs from miscanthus reported by Yan et al. [50]. Thanks to using an unorthodox source of carbon, the authors needed to employ additional equipment to process the raw material, such as a tubular furnace and planetary ball mill. When synthesizing more complex nanoparticles or nanocomposites, the sonochemical methods are often used in tandem with conventional approaches such as hydrothermal methods or calcination [47,48,51]. These methods also require the use of specialized equipment such as autoclaves. A great example of such synthesis can be found in the work of Jiabei Wang and Zhiqiang Jiang in which the authors synthesized a series of porous S-doped carbon nitride ribbons (PSCN) (see Figure 12) [50].

First, a solution of melamine (2 g) in 60 mL of glycerol was prepared. Then, 0.8 mL of concentrated sulfuric acid was added and stirred for 15 min. Afterwards, the mixture was heated in an autoclave at 150 °C for 12 h and subsequently filtrated. Next, to the slurry obtained after filtration, 20 mL of ethanol and 20 mL of deionized water were added, and the resulting mixture was ultrasonicated at 80 °C for 1 h. Then, it was once again filtered, and the precipitated solid was washed with ethanol and deionized water, which was followed by drying for 24 h at 80 °C in a vacuum oven. Finally, PSCN was obtained by calcinating the resulting solid at 550 °C for 2 h with a heating rate of 2 °C⋅min^−1^.

**Figure 13:**
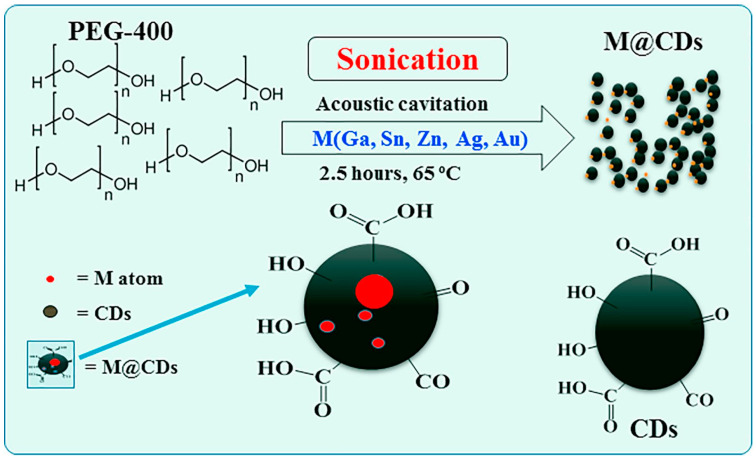
The synthesis process and possible formation mechanism of metal-doped carbon dots (M@CDs). Reproduced under the Creative Commons license from ref. [45].

The possibilities that sonochemical methods give allow not only for the synthesis of pure carbon nanomaterials but also enhancing the desired properties which those materials exhibit. A variety of different modifications can also be made with the addition of nitrogen atoms into the carbon nanomaterial, of which one is called doping [40,42] (Figure 13). Other modifications that can be achieved with the help of ultrasound techniques include doping nanomaterials with different kinds of metals, such as gallium [52], tin [53], or palladium [44], and non-metals such as nitrogen [42] or sulfur [51] and combining carbon nanomaterials, such as carbon quantum dots (CQDs) with semiconducting metal oxides such as rutile or anatase TiO_2_ [54] and SnO_2_ [43].

### 3.2. Sources of Carbon Used for Sonochemical Synthesis

**Figure 14:**
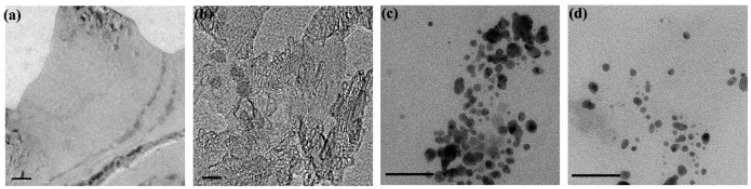
TEM images of GO-like carbon flakes (**a**) before sonication (**b**) after sonicating for 5 min, (**c**) after sonicating for 15 min, (**d**) after sonicating for 60 min (Scale bars indicate 50 nm). Reproduced under the Creative Commons license from ref. [49].

By utilizing different sources of biomass such as crab shells, onion sheathing, or miscanthus [40,50], it is possible to create distinct types of carbon nanomaterials with different properties. By using waste onion sheathing [49], the authors were able to create graphite oxide (GO)-like flakes which were then turned into their nano-sized counterparts with the help of ultrasonic treatment (Figure 14). This method allowed for a cost-effective, green synthesis of materials which could then be potentially used as drug carriers and radioprotective agents. The use of crab shells as a carbon source proved to be an effective way to obtain carbon dots doped with nitrogen atoms (N@CQDs) [40]. In the literature, there are also reports of strategies that allow the synthesis of one-dimensional carbon nanorods and two-dimensional graphene nanoribbons from previously made metal–organic frameworks (MOFs) with the help of a sonochemical treatment called sonochemical exfoliation [55].

Other, more conventional reagents that are used for the preparation of carbon nanoparticles (CNPs) include polyethylene glycol [39,52], graphene [54] or graphene flakes [47], and glucose [38,41].

### 3.3. Effect of Synthesis Conditions on Properties of Produced Nanoparticles

In the past few years, different sonochemical procedures have been reported, which allow the synthesis of carbon nanomaterials of various properties from a wide range of raw materials and reagents. These procedures are characterized by their relatively short duration [39,44,47,50,52], ranging from 60 to 180 min, with only a couple of them taking significantly longer [38]. While allowing for shorter synthesis times, it was shown that lowering the reaction time to 30 min caused a significant decrease in the quality of obtained QDs or even the inability to obtain any at all [39,52]. The effect of longer sonication times was also tested, and it was different depending on the type of synthesized nanoparticles. In the case of gallium-doped CQDs (Ga@CQDs), the authors [52] stated that a “longer sonication time (180 min) led to smaller fluorescence intensity”, while for non-doped CQDs, such effect was only slight [39]. For the synthesis of graphene nanosheets, the sonication time also plays a crucial role. It was stated that a sonication time of 120 min of continuous ultrasonic treatment caused the yield of graphene nanosheets to decrease [47]. Other crucial factors in this type of synthesis are the initial sonication temperature and amplitude. It was proven [39] that depending on the sonication amplitude, a visible change in the emission intensity of the nanoparticles occurs, which is stated to be caused by changes in the size distribution and possibly the number of nanoparticles in the prepared suspensions. The effect of different initial temperatures was similar to the one observed for different sonication times. Lower temperature (6 °C) caused an absence of fluorescence, while at 25 °C, fluorescent bands were significantly lower than for higher temperatures (55, 65, and 75 °C), and the higher the temperature, the more intense fluorescence was observed.

With the use of sonochemical methods, it is also possible to acquire high yields of synthesized nanoparticles. Depending on the type of nanoparticles that were synthesized, different modifications to the process of synthesis were made. For the preparation of carbon nanotubes, in addition to the initial sonochemical treatment, a hydrothermal method was employed, which resulted in a 70% yield of carbon nanotubes from the original reagents used for synthesis [48]. A high yield (>75%) of different carbon nanoparticles, mainly graphene nanoribbons, was achieved by K^+^-assisted sonochemistry, which proved to be higher than those that were prepared by the exfoliation method [55].

Sonochemically synthesized carbon nanoparticles are reported to be stable in different operations that they can be used for, such as catalysis [46], fluorescent-based detection [42], and electrochemistry [43].

Their efficiency in terms of photoluminescence is often described by a number called quantum yield (QY), which is defined as a number of photons emitted by a system for every photon that the system has absorbed. CQDs synthesized with the help of sonochemical methods can achieve a QY of up to 20% [53]. For pure CQDs, the QYs are reported to be around 16% [39]. It is possible to increase the efficiency of these nanomaterials by doping them with tin atoms [45,53], while by adding different metals, the quantum yield tends to drop [45] (Table 4).

**Figure 15:**
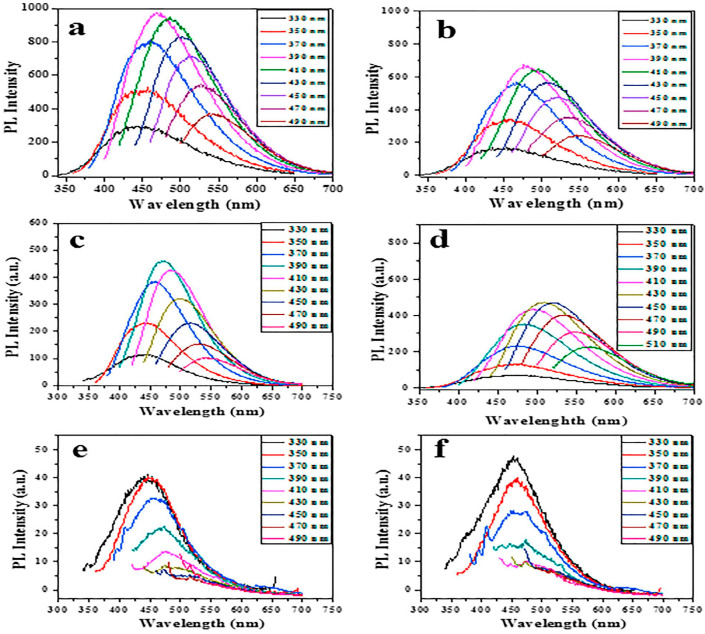
Fluorescence spectroscopy of sonochemically synthesized (**a**) CQDs, (**b**) Ga@CQDs, (**c**) Zn@CQDs, (**d**) Sn@CQDs, (**e**) Ag@CQDs and (**f**) Au@CQDs at different excitation wavelengths. Reproduced under the Creative Commons from ref. [45].

Depending on the type of nanoparticles synthesized, their maximum fluorescence peaks can appear at different wavelengths. For example, while typical emission peaks for pristine CQDs tend to appear at 440–460 nm, the peak for Ga@CQDs appeared at 416 nm when excited with 390 nm photons (Figure 15) [52].

### 3.4. Applications of Sonochemically Synthesized Carbon Nanomaterials

As previously stated, carbon nanomaterials have found many applications in different fields. Graphene nanoribbons, for example, are proven to exhibit excellent supercapacitor performance [55] and Sn nanoparticles decorated with Sn@CQDs showed promising anode activities in Li-ion batteries [53]. When CQDs were combined with lanthanoid metal–organic frameworks (Ln-MOFs), researchers managed to obtain white light-emitting diodes (WLEDs) with high color rendering [56].

Ga@CQDs are reported to generate more reactive oxygen species in the form of singlet oxygen than their non-modified counterparts, and because of that, there exists a possibility of employing them in photodynamic therapy [52]. N@CQDs can also be used in the medical field as an effective imaging agent in cancer diagnosis due to their high fluorescence and biocompatibility [40]. They also exhibit a significant decrease in fluorescence with the presence of Fe^2+^ ions, which could be used for the detection of said ions [42]. Other noteworthy carbon nanomaterials that could find their use in the medical field due to their useful properties combined with excellent biocompatibility and low toxicity include GO-like carbon flakes [49] and zinc sulfide nanoparticles loaded on reduced graphene oxide (ZnS/NPs@rGO) [57].

Other applications of different kinds of carbon nanomaterials include electrocatalysis [43,47], photocatalysis [38,51,54], and H_2_O_2_ production (Table 5) [46]. The recent literature also suggests that nanocomposites of GO and palladium could have the potential to be an efficient material for hydrogen storage with a storage capacity of 5.09 wt. % [44].

## 4. Sonochemical Synthesis of Oxide Nanostructures and Their Applications

The general procedure for the sonochemical synthesis of oxide nanomaterials is the following: a precursor solution is mixed with a precipitating agent and treated with ultrasound; then, products are separated, washed, dried, and optionally calcined [58]. Each step of this process can be modified to achieve the desired properties of the material. For example, Teh et al. reported that an increase in ultrasound intensity accelerates the growth and crystallization of titania nanoparticles [59]. This conclusion is consistent also with the research Saravanan et al. conducted on CuO [60]. Taunk and Singh have also demonstrated a one-pot sonochemical synthesis of CuO nanoparticles in aqueous solution without the usage of surfactants, templates, or organic solvents [61]. Karizi et al. analyzed the influence of initial zinc acetate concentration on a [Zn_2_(btec)(DMF)_2_]_n_ metal–organic framework [62]. The results demonstrated an increase in particle size with an increase in the initial concentration of reagents; moreover, low concentrations caused the formation of rod-like structures. NPs have a big surface area and consequently surface tensions, which led to the formation of agglomerates and superstructures. Bao et al. examined the influence of solvent in the sonochemical synthesis by providing a reaction in mixtures consisting of ethylene glycol and water with different ratios from 10 to 100% *v*/*v* [63]. Investigation resulted in a variety of superstructures: sphere-like, twin-sphere-like, bouquet-like, and hexagonal-prism-like. The authors connect morphology with the amount of [Zn(OH)_4_]^2−^ depending on the solvent content. Moghtada et al. synthesized CoTiO_3_ using the sonochemical method and observed that the addition of EDTA to the reaction mixture causes the formation of agglomerates with a more homogenous structure [64]. Interestingly, magnetic nanoparticles of magnetite (Fe_3_O_4_) and magnetite/silica nanocomposites (Fe_3_O_4_/SiO_2_) were possible to achieve sonochemically, exhibiting narrow size distributions of c.a. 10 nm and 100 nm, respectively [14]. Moreover, the authors speculated that the shock waves resulting from acoustic cavitation had a protective impact on the surface of nanoparticles [14].

### 4.1. Sonochemically Synthesized Oxide Nanomaterials for Catalysis

Catalysis plays a big role in various technological processes. Ultrasound cavitation is an excellent method for the synthesis of nanoparticles with demanded features by controlling sonochemical effects. Bhosale et al. received Cu_2_O as an efficient catalyst for the coupling reaction of N-arylation of imidazole [65]. For such synthesis, copper acetate and glycerol were mixed and sonicated for 1 h. Afterwards, the reaction mixture was diluted, centrifuged, and washed with water and ethanol. The obtained Cu_2_O NPs were characterized by XRD, TEM, EDS, FT-IR, and BET surface area measurement, and then the catalytic activity of the coupling reaction of amine and aryl halide was determined [65]. It was reported that the BET specific surface area of copper oxide nanoparticles was 17 m^2^/g, while commercial material showed only 0.76 m^2^/g. The XRD pattern is consistent with the literature data, the average grain size of Cu_2_O was calculated by Scherrer’s equation and found to be 12 nm. TEM analysis showed that the spherical morphology and measured sizes of particles were in the range of 80 to 150 nm. A catalytic coupling reaction between aryl halide and amine was conducted, the yield was up to 98%, and the results presented a significantly higher activity of sonochemically synthesized particles in comparison to those commercially available [65].

Abbas et al. developed a sonochemical synthesis of Au-decorated Fe_2_O_3_/ZrO_2_ nanocubes as a catalyst for Fischer–Tropsch synthesis [66]. To obtain Fe_2_O_3_/ZrO_2_ nanoparticles, a water solution containing FeSO_4_ and ZrOCl_2_ was placed in an ultrasonic chamber; then, ethanol and propanol were added. The resulting mixture was sonicated for 50 min and calcined at 600 °C for 2 h. For decorating the obtained nanocomposite with gold, HAuCl_4_ was added to the Fe_2_O_3_/ZrO_2_ dispersion in water solution with pH 10 adjusted by ammonia solution, and the mixture was sonicated. It was found that the Fe/Zr molar ratio strongly influences the morphology and catalytic properties of the material. An increase in iron content causes an increase in the selectivity of moderate C5+ products, while an increase in Zr content leads to deactivation. The selectivity of FTS products shifted from a high CH₄ ratio of 75% using Fe₂O₃/ZrO₂ nanocubes (with a molar ratio of 1) to a high liquid hydrocarbons selectivity of 79.5% when 2.5 wt. % Au was coated on the Fe₂O₃/ZrO₂ catalyst. This enhancement in catalytic activity after doping with Au nanodots is attributed to improved dispersion and reducibility, inhibition of the hydrogenation process on the Fe active sites, and a significant increase in catalyst pore size [66].

**Figure 16:**
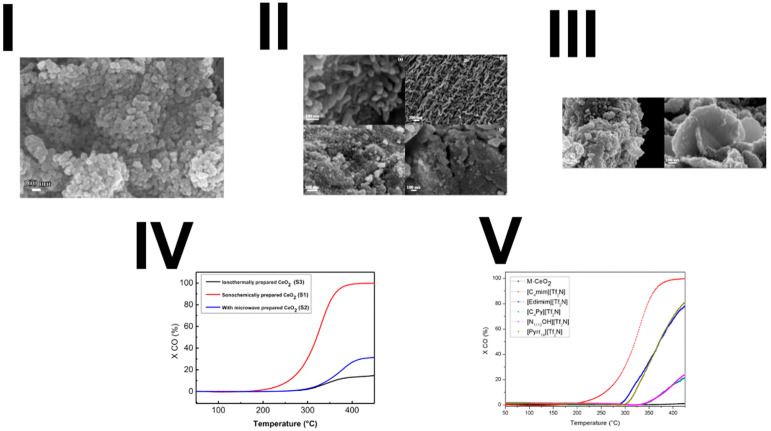
(**I**) SEM image of CeO_2_ prepared via sonochemical synthesis with NH_4_OH as the precipitator, (**II**) SEM images of as-obtained CeO_2_ in (**a**) [Edimim][Tf_2_N], (**b**) [Pyrr_14_][Tf_2_N], (**c**) [C_4_Py][Tf_2_N], and (**d**) [N_1112_OH][Tf_2_N], (**III**) SEM images of sonochemically prepared CeO_2_ nanoparticles from cerium(III) nitrate hydrate (left) and CeO_2_ nanosheets prepared from cerium(III) chloride hydrate (right), (**IV**) CO oxidation over CeO_2_ obtained by different preparation methods, (**V**) catalytic activity for CO oxidation of CeO_2_ prepared in different ionic liquids in comparison with bulk ceria (M-CeO_2_). Reproduced with consent from [67].

Alammar et al. received CeO_2_ for CO oxidation using an ionic-liquid-assisted sonochemical route [67]. In this synthesis, cerium(III) salt was mixed with ionic liquid and NaOH in one synthesis and NH_3_ in the other to examine the effect of the precipitating agent. The mixture was stirred for 30 min and then treated with ultrasound for 12 h. The product was isolated by centrifugation, washed with ethanol and water, and dried. The results of the sonochemically synthesized material were compared to ionothermal and microwave-assisted methods. The morphology of particles was different (images are provided in Figure 16): nanorods with a size of 20 nm in diameter and 150 nm length in the case of [Edimim]^+^, and nanospheres using [C_4_mim]^+^, [C_4_Py]^+^ and [N_1112_OH]^+^ with an average crystal size of less than 10 nm. In addition to this, the usage of cerium chloride or nitrate instead of acetate led to CeO_2_ nanosheets. The effect of precipitator (NH_3_ instead of NaOH) resulted in a bigger particle size and lower BET surface area. The catalytic performance for the CO oxidation of samples was investigated (see Figure 16), and the highest activity showed CeO_2_ prepared from cerium acetate with NaOH as the precipitating agent and ionic liquid [C_4_mim][Tf_2_N]. The author reported that it could be explained by the high density of the surface oxygen vacancies resulting from the reduction of Ce^4+^ to Ce^3+^, which was verified by UHV-FTIRS data.

### 4.2. Sonochemically Obtained Oxide Nanomaterials for Photocatalysis

Large-scale production is tightly connected with the release of toxic organic pollutants. Photocatalysis is one of the economical methods for the treatment of wastewater using solar energy. This technology is one of the advanced oxidation processes. Photo-induced electrons and electron holes react with water, producing hydroxyl radicals ·OH and superoxide radical anions ·O^2−^, which oxidize organic pollutants in wastewater. Many researchers try to improve the solar efficiency of sonochemically synthesized oxides by adding dopants to their materials or using composites to apply the heterojunction effect.

**Figure 17:**
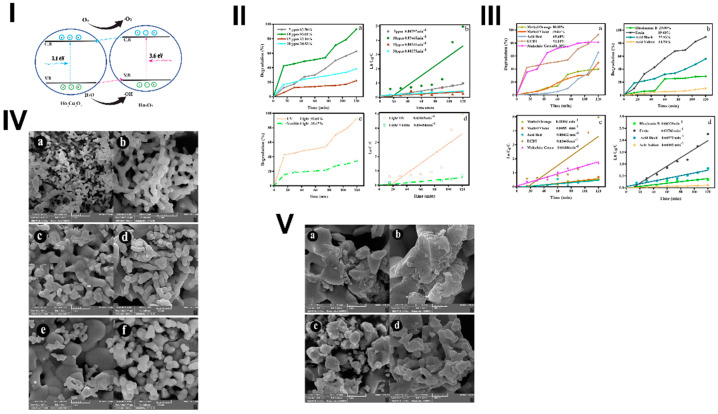
(**I**) Scheme of photocatalytic degradation mechanism of Ho_2_Cu_2_O_5_/Ho_2_O_3_ nanocomposites, (**II**) photocatalytic performance and kinetic linear simulation plots of Ho_2_Cu_2_O_5_/Ho_2_O_3_ nanocomposites under (**a**,**b**) different dye concentrations (5, 10, 15, 20 ppm) and (**c**,**d**) different light sources, (**III**) photocatalytic activity and kinetic linear simulation plots of Ho_2_Cu_2_O_5_/Ho_2_O_3_ nanocomposites for removal of (**a**,**b**) Methyl Orange (MO), Methyl Violet (MV), Acid Red (AR), Eriochrome Black T (ECBT), Malachite Green (MG); (**c**,**d**) Rhodamine B (RhB), Eosin (EO), Acid Black (AB), and Acid Yellow (AY) after 120 min UV illumination; (**IV**) the SEM images of Ho_2_Cu_2_O_5_/Ho_2_O_3_ nanocomposites prepared with a power of 45 W for (**a**,**b**) 10 min (**c**,**d**) 20, and (**e**,**f**) 30 min, (**V**) the SEM images of Ho_2_Cu_2_O_5_/Ho_2_O_3_ nanocomposites prepared with a power of 45 W for 10 min at sonication time pulse (**a**,**b**) 0.1 s and (**c**,**d**) 0.5 s. Reproduced under the Creative Commons license from ref. [68].

Tabatabaeinejad et al. received mixed holmium copper oxide [68]. Holmium(III) and copper solutions with a molar ratio of 1:1 were mixed together and sonicated with different sonication times and ultrasound pulse times. Then, TEPA was added dropwise until pH 10. Next, the resulting mixture was stirred at 80 °C for 20 min. Finally, the gel-like product was separated, dried, and calcined at 700 °C and 1000 °C. Figure 17IV demonstrates SEM images of products with different reaction times. The authors suggest that the optimal time of sonication is 10 min; within that time, more porous agglomerates are formed. Figure 17V provides images of particles sonicated for 10 min but at different pulse rates with longer pulse rates causing the formation of uniform morphology. Two bandgaps were calculated with the Tauc method: 3.6 eV and 3.1 eV for Ho_2_O_3_ and Ho_2_Cu_2_O_5_, respectively. The value of bandgaps allows for a heterojunction between Ho_2_Cu_2_O_5_ and Ho_2_O_3_, which significantly impacts the photocatalytic properties of the obtained material (the scheme is provided in Figure 17I). Photocatalytic experiments were conducted with different dyes and sources of light (Figure 17II,III). It appeared that the highest efficiency was obtained against Eriochrome black T (ECBT) photodegradation. After 2 h of ultraviolet irradiation, 93% of the dye was removed; this reaction was pseudo-first-order kinetics with the rate of 0.03465 min^−1^ [68].

Lee et al. synthesized Ce-doped TiO_2_ by the following method: cerium nitrate and surfactant P-123 were dissolved in water, and then titanium isopropoxide was added dropwise while stirring [69]. The mixture was then sonicated for 1 h and followed by aging for 12 h. The resulting product was centrifuged, washed, and dried at 80 °C for 12 h. The last step was the calcination of powder at 500 °C for 1 h. The study revealed that the addition of cerium affects the particle size and specific surface area. Photocatalytic experiments showed that the addition of cerium improved catalytic properties, and the best performance of the degradation of o-xylene was achieved with a 0.75% weight ratio of cerium [69].

Shende et al. developed a sonochemical synthesis of graphene–TiO_2_ composite with Ce and Fe doping [70]. TEM images showed that the size of TiO_2_ nanoparticles was lower than 10 nm, and ultrasound cavitation caused a uniform distribution of particles on graphene nanosheets. The determined BET surface area of graphene–Fe–TiO_2_, graphene-Ce–TiO_2_, and graphene–TiO_2_ nanocomposites is 16.60, 4.66, and 2.58 m^2^/g. The addition of Ce and Fe improved photocatalytic performance—the comparison has been made between graphene–TiO_2_, graphene–Ce–TiO_2_, and graphene–Fe–TiO_2_ catalysts for the condition of initial dye concentration of 60 ppm, pH 6.5, and catalyst loading of 6 mg. The rate constants for pure graphene–TiO_2_, graphene–Fe–TiO_2_, and graphene–Ce–TiO_2_ are, respectively, 0.0111 min^−1^, 0.0161 min^−1^, and 0.0129 min^−1^.

Khataee et al. doped zinc oxide with holmium by adding NaOH to a solution containing ZnCl_2_ and Ho(NO_3_)_3_ until the pH reached 10; afterwards, the mixture was sonicated for 3 h and then washed and dried [71]. SEM investigation demonstrated that the incorporation of Ho into the ZnO crystal lattice prevents aggregation. The degradation efficiency increased with the addition of a dopant, and 4% Ho-doped ZnO NPs were chosen as the most effective catalyst. A series of experiments with different radical scavengers revealed that free radicals are the dominant controlling mechanism for the sonochemical degradation of Reactive Orange 29 [71].

Amulya et al. synthesized mixed iron manganese oxide [72]. MnSO_4_ and FeSO_4_ with a molar ratio of 1:2 were dissolved in water, the pH was adjusted to 14 with NaOH, and the mixture was sonicated for 2 h. After the reaction product was filtered, washed, dried, and then calcined at 650 °C for 2 h. The structure was confirmed by XRD and EDX analysis. The size of nanoparticles was calculated with Scherrer’s equation and confirmed by TEM—it was reported to be 25 nm [72]. The obtained material demonstrated high photocatalytic activity in the degradation of cationic dye Methylene Blue, achieving a degradation percentage of 96%. In contrast, under the same conditions, the degradation of anionic dye, Drimaren Yellow, was only 7%.

Panahi et al. sonochemically synthesized nanorods of thulium vanadate (TmVO_4_) using Schiff bases as ligands [73,74]. Synthesized nanoparticles exhibited a specific surface area of 24.91 m^2^/g and an energy bandgap of 2.3 eV, showing potential application in photocatalysis [73]. The authors demonstrated the utilization of TmVO_4_ nanorods as photocatalysts for the removal of Methyl Violet and Eriochrome Black T, and the degradation efficiency reached more than 97% [73].

Mahdi et al. used a sonochemical method to obtain holmium stannate NPs of stoichiometry Ho_2_Sn_2_O_7_, which exhibited a relatively high energy bandgap of value 3.9 eV, making this nanomaterial suitable for photocatalysis in the UV region [75]. The authors achieved a relatively high degree of photodegradation of distinct dyes (e.g., 95% for Eriochrome Black T), and the experiments with scavengers confirmed that the process is mediated mainly by superoxide radicals and holes [75]. The influence of sonication time and ultrasound power was also investigated, and it was found that a decrease in sonication time from 15 to 10 min favors an agglomeration to some degree [75]. The same trend was observed while increasing the ultrasound power from 60 to 80 W [75].

Sonochemically synthesized Sm_2_CuO_4_ nanostructures showed an optical bandgap of 1.62 eV and were utilized as photocatalysts in the degradation of Methyl Orange with 91.4% degradation [76]. The positive holes were the main mediator in the process [76].

### 4.3. Sonochemically Obtained Oxide Nanomaterials for Toxic Metal Remediation

Toxic metals are widely used in industry, agriculture, medicine, and technology, leading to their increased spread in the environment. Contamination by dangerous elements poses a significant threat to human health, making the remediation of toxic elements a vitally important issue.

Yadav et al. produced iron oxide nanoparticles for the remediation of heavy metals from water [77]. For nanomaterials, synthesis solutions of FeCl_3_ and FeSO_4_∙7 H_2_O with a molar ratio of 2:1 were mixed and sonicated at 60–70 °C. Then, NaOH was added dropwise to obtain pH 11–13; after the formation of black precipitate, the addition of NaOH was stopped. The flask was closed and sonicated for 1 h. After the reaction, the mixture was cooled, centrifuged, washed, and dried. Studies showed that the obtained iron oxide nanomaterials can be used for heavy metals removal from water solutions after 12 h efficiency, which was 97.96% and 82.8% for Pb^2+^ and Cr ions, respectively. Also, it was reported that amorphous nanoparticles can be converted into the crystalline phase by annealing [77].

Parveen et al. developed a synthesis of aluminum hybrids for remediation [78]. Aluminum oxide was synthesized from aluminum nitrate with ammonium solution as a precipitating agent using a sol–gel-assisted sonochemical route. The obtained precipitate was washed, dried, and calcined at 500 °C for 4 h. The obtained product was modified using a solution of 3-aminotriethoxypropylsilane (APTES) in isopropanol. Then, amine-functionalized aluminum oxide was dispersed in dichloromethane and indole or indole derivatives were added; the resulting mixture was sonicated for 1 h. For comparison, indole was also added without further sonication, resulting in total of six products for aluminum oxide functionalized with indole, indole-2 carboxylic acid, 2-methyl indole, and their non-sonicated versions. SEM images of sonicated and non-sonicated samples were compared. It appeared that ultrasound provided the formation of spherical-shaped clusters and the formation of porous structures. In addition, indole derivatives caused the formation of additional reactive points. Adsorption analysis showed that the optimal conditions for lead and mercury removal are reached with pH 7 and a contact time of 1 h. Ultrasound-treated products demonstrated better adsorption of up to 67% for lead and 63% for mercury in comparison to non-sonicated aluminum hybrids 50% and 58%, respectively [78].

### 4.4. Sonochemically Obtained Oxide Nanomaterials for Sensor Applications

Sensors can be divided into two main groups: enzymatic and non-enzymatic. Enzymatic sensors are well researched, but they have critical drawbacks such as a strong dependence of signal on pH, concentration of O_2_, temperature and humidity. Thus, non-enzymatic sensors are being developed, and sonochemically synthesized oxide nanomaterials have great potential for producing sensors with a wide linear range of concentrations and low detection limits.

**Figure 18:**
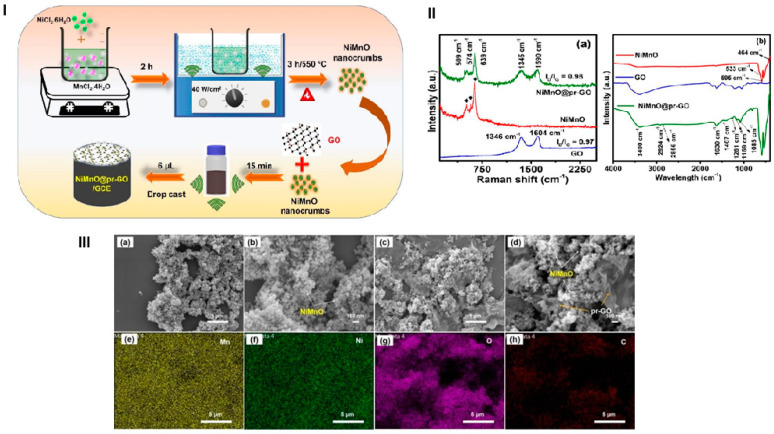
(**I**) Scheme of sonochemical synthesis of NiMnO@pr-GO nanocomposite. (**II**) (**a**) X-ray diffraction pattern of NiMnO nanocrumbs and NiMnO@pr-GO nanocomposite. (**b**) EIS of bare/GCE, GO/GCE, NiMnO/GCE, and NiMnO@pr-GO/GCE. (**III**) FE-SEM images of NiMnO nanocrumbs (**a**,**b**) and NiMnO@pr-GO composite (**c**,**d**) and corresponding elemental mapping of NiMnO@pr-GO (**e**–**h**). Reproduced under the Creative Commons license from ref. [79].

Vivekanandan et al. worked on the synthesis of partially reduced graphene oxide decorated with NiMnO (NiMnO@pr-GO) [79]. For NiMnO manufacturing, the following procedure was adopted (see Figure 18): nickel and manganese(II) chlorides were dissolved in water; then, the pH was adjusted to 10 with NaOH, and after 2 h of sonication, washing, and drying, the obtained material was calcined at 550 °C for 3 h. For nanocomposite synthesis, GO powder and NiMnO was mixed in ethanol and placed in an ultrasonic bath for 15 min followed by drying. For electrochemical investigations, the glassy carbon electrode (GCE) was coated with the obtained composite using the drop-casting method. The scheme of the synthesis is shown in Figure 18I.

The obtained composite was characterized by SEM; the images show a NiMnO crumb-like nanostructure. The XRD analysis presented in Figure 18II revealed that the obtained NiMnO consists of two phases, NiMnO_3_ and NiMn_2_O_4_. The electrochemical analysis of metronidazole detection demonstrated that the NiMnO@pr-GO/GCE showed a wider linear range of concentrations from 0.1 to 234 µM. Moreover, the mentioned electrode exhibited good selectivity, sensitivity, and applicability in terms of MNZ sensing.

Chen et al. synthesized TiO_2_-decorated graphene oxide for the detection of theophylline [80]. TiO_2_ microspheres were produced by the sonication of titanium(IV) isopropoxide in ethylene glycol solution; then, the precipitate was filtered, washed, and calcined at 600 °C. Then, the obtained titania powder was sonicated with graphene oxide in the ethanol–water mixture to receive a TiO_2_@GOS (graphene oxide sheets) composite. FESEM images revealed the uniform size and shape distribution of the synthesized composite with an average crystallite diameter of 60 nm. Glassy carbon electrodes coated with TiO_2_@GOS showed reproducible responses with a wide linear range of concentrations (0.02 to 209.6 µM) and an excellent detection limit of 13.26 nM [80].

Elshikh et al. received a MnFe_2_O_4_/C_3_N_4_ composite for serotonin detection [81]. For the synthesis of MnFe_2_O_4_ iron(III) and manganese(II), salts were dissolved in water, and after the addition of KOH, the resulting mixture was sonicated with the subsequent calcination at 450 °C. Then, 10 mg of MnFe_2_O_4_ was mixed with 5 mg of carbon nitride (C_3_N_4_) and added into water with further sonication to create MnFe_2_O_4_/C_3_N_4_. For electrochemical investigation, a glassy carbon electrode was coated with the drop-casting method. An analysis of TEM images showed that carbon nitride sheets were decorated with spherical particles of mixed iron manganese oxide. The average particle size of MnFe_2_O_4_ is 216 nm, and the purity was analyzed with XRD and XPS. Electrochemical investigation revealed the high performance of the detection of serotonin in biological samples, and the large surface area caused a good electron transfer rate. The sensor based on the synthesized MnFe_2_O_4_/GCN composite demonstrated excellent detection limits in both human and rat serum [81].

### 4.5. Sonochemically Obtained Oxide Nanomaterials for Capacitors

Nanoporous materials have a high surface area and consequently ion transport, resulting in high capacitive properties. Therefore, sonochemical oxide nanostructures can be used as relatively cheap materials with high energy density and specific capacitance.

Naderi et al. produced Co_3_O_4_—RGO (reduced graphene oxide) and Co_3_O_4_/NRGO (nitrogen-doped reduced graphene oxide) nanocomposites [82]. Particles were prepared by mixing RGO or NRGO with cobalt acetate water solution followed by 30 min of sonication with the further addition of NH_4_OH. For electrochemical studies, a steel grid was coated with mixtures of the 75:15:5 (wt./wt./wt.) prepared materials, carbon black and poly(tetrafluoroethylene). XRD, Raman, FE-SEM, and XPS were used to confirm the structure of the obtained product. The special capacitance value of Co_3_O_4_/NRGO reached 763 F/g at a 2 mV/s scan rate. The Co_3_O_4_/NRGO-based electrode demonstrated good stability: after 4000 cycles, the special capacitance was 98.9% of the start value. A comparison to Co_3_O_4_/RGO revealed that nitrogen doping is an effective method for improving capacitance properties [82].

Duraisamy et al. produced nanostructured NiO [83]. For synthesis, NaOH was added to NiCl_2_ water solution and sonicated for 1 h. The obtained precipitate was calcined at three different temperatures, 250, 450, and 650 °C, and the obtained products were named N1, N2, and N3, respectively. To produce a working electrode, 75% wt. nanoparticles were mixed with 15% acetylene black and 10% of PVDF in NMP and were drop cast on nickel foam and dried. Electrochemical experiments were conducted using a three-electrode system where NiO-coated nickel foam was the working electrode, the AgCl/Ag electrode was the reference electrode, and platinum wire was the counter electrode. Investigations were performed in 1 M KOH electrolyte. Microscope analysis revealed that an increase in calcination temperature has a significant impact on particle size. The average particle sizes are 6 nm, 21 nm, and 41 nm, corresponding to N1, N2, and N3, respectively. It turned out that bigger particles have a lower specific capacitance, and electrochemical experiments revealed that the best performance is shown by N1 with a specific capacitance of 449 F/g at a scan rate of 5 mV/s. The obtained material showed 74% stability after 500 cycles. After 4 years, Numan et al. manufactured Co_3_O_4_ porous 2D nanoflakes in a similar synthesis [84]. The obtained particles have an average size in the range of 200–250 nm with a pore size of less than 20 nm. The specific capacity value was 104.4 C/g, while the maximum energy density and power density were 23.7 Wh/kg and 307 W/kg, respectively [84].

Raj et al. used sonochemical synthesis to produce a cathode material (CuCo_2_O_4_) and anode material (nanocomposite of FeWO_4_ and reduced graphene oxide) with respective maximum capacitance values of 356.6 and 414 F/g [85].

On the other hand, Sun et al. were able to sonochemically prepare ZnCo_2_O_4_ in the form of two distinct nanostructures varying the pH [86]. Under pH 7.5, the resulting product was in the form of nanoparticles, while the product obtained under pH 9.5 was in the form of chain-like nanostructures [86]. The ZnCo_2_O_4_ NPs obtained under pH 7.5 had a greater specific surface area (148.77 m^2^/g) and better electrochemical properties than in the case of ZnCo_2_O_4_ nanostructures synthesized under pH 9.5 [86].

### 4.6. Sonochemically Obtained Oxide Nanomaterials for Medical Applications

Ultrasound cavitation can be employed to create oxide nanostructures for innovative use in medicine exactly as an antimicrobial coating, cancer treatment, and dental surgery.

**Figure 19:**
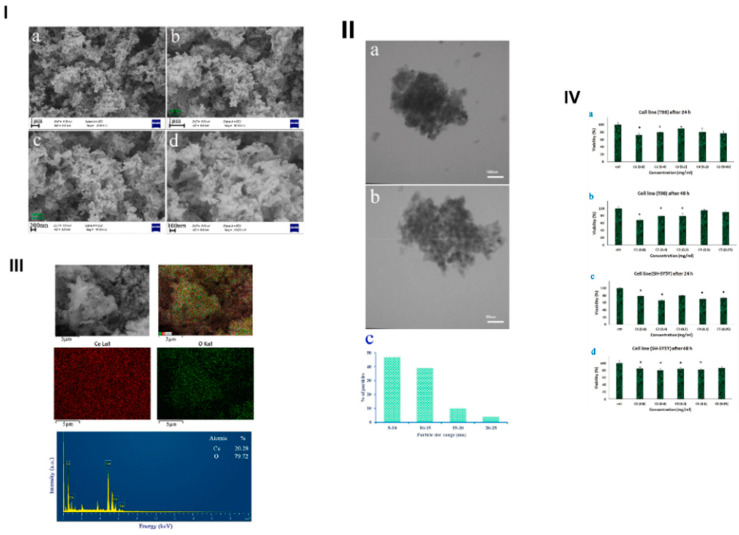
(**I**) FESEM images of the nanostructured CeO_2_ fabricated using Mentha extract, in different magnifications. (**II**) (**a**,**b**) TEM images and (**c**) typical histogram of the particle sizes of the nanostructured CeO_2_ fabricated using Mentha extract. (**III**) FESEM image, EDS elemental mapping images, and EDS graph of the nanostructured CeO_2_ fabricated using Mentha extract. (**IV**) In vitro cell viability assay, cell viability of T98 cell lines incubated with cerium dioxide NPs at different concentrations for 24 h (**a**) and 48 h (**b**) and in vitro cell viability assay, cell viability of SHSY5Y cell lines incubated with cerium dioxide NPs at different concentrations for 24 h (**c**) and 48 h (**d**). Mean ± SD of five independent experiments, each performed in duplicate. Star indicates the value is significantly different from the control untreated cells (*p* < 0.05). *p*-values less than 0.05 were considered significant. Reproduced under the Creative Commons license from ref. [87].

Zinatloo-Ajabshir et al. researched sonochemically obtained CeO_2_ in terms of cancer treatment. For cerium dioxide synthesis, cerium(III) nitrate was dissolved in water with the addition of Mentha extract as a capping agent; afterwards, the mixture was sonicated for 12 min with the simultaneous addition of ammonia [87]. The next step was washing and drying of the gel-like precipitate. Finally, the product was calcined at 500 °C for 2 h. SEM images (Figure 19) present the agglomeration of spherical nanoparticles into sponge-like structures. It proves that Mentha extract can be used as a capping agent to create a desirable morphology. The purity of cerium oxide was confirmed with EDS. Cytotoxic in vitro investigation conducted on cell lines (T98 and SHSY5Y) with different nanoparticle concentrations and exposure times of 24/48 h showed that CeO_2_ can be used for cancer treatment; however, more studies are needed.

Svirinovsky et al. examined the hydrophobic and antibacterial properties of ZnO and CuO-coated polyethylene sheets [88]. Oxides were obtained by the sonication of acetate salt in water–ethanol (1:9 by volume) solution at 60 °C with ammonia as the precipitating agent. SEM images showed that the size of the obtained ZnO particles was in the range of 110–170 nm, while the size of the CuO particles was about 50 nm. The produced coating demonstrated excellent hydrophobic properties with a water contact angle of 160° compared to 100° in terms of uncoated polyethylene. In addition, both CuO and ZnO completely killed *S. aureus* bacteria. The authors attribute this phenomenon to the generation of reactive oxygen species, which was the result of contact of water with nanoparticles [88].

Stubbing et al. received ZnO and CuO nanoparticles from acetate salts with ultrasound generated by dental instruments [89]. Such synthesized copper and zinc oxides were tested on different tooth models and proved to have potential use in disinfection during some kind of dental surgery operations [89].

Noman et al. developed a sonochemical synthesis of ZnO on cotton in situ [90]. At first, cotton was immersed in distilled water and then ZnCl_2_ was added; after, the pH was adjusted to 9 with NaOH. Next, the solution was sonicated, and then it was washed and dried. Eight versions of synthesis were carried out where the zinc chloride amount was 10 g or 20 g, the reaction time was 60 and 120 min, and the horn intensity was 50% and 70%. The biggest amount of deposited ZnO was reached with 20 g ZnCl_2_, 120 min reaction time, and 50% horn intensity, while the best ultraviolet protection was in the case of 10 g ZnCl_2_, 60 min reaction, and 50% horn intensity [90]. XRD analysis revealed that the obtained zinc oxide was pure with an average particle size in the range of 26.8–28.2 nm. The ZnO cotton composite was reported to show high antimicrobial activity. Moreover, the obtained material was stable after washing, and the tensile strength before and after synthesis remained the same, which confirms potential use in industry.

The overview of applications, as well as procedures of sonochemical syntheses, for oxide nanostructures derived with the assistance of ultrasound is presented in Table 6.

## 5. Sonochemical Synthesis of Chalcogenide Nanostructures and Their Applications

Chalcogenides are materials that consist of metal atoms and chalcogens—S, Se, and Te. Although similar in chemical composition to oxide materials (chalcogens form analogical anions to O^2−^, that is S^2−^, Se^2−^, and Te^2−^), chalcogenide materials exhibit often unique properties. This is caused by their relatively higher electron density, which contributes to, for example, stronger van der Waals forces and results in the formation of unique 2D materials not seen in the case of oxides. The chemistry of chalcogenides is also more diverse than that of the oxides because chalcogenides are prone to oxidation and hydrolysis. Thus, care must be taken during the synthesis of chalcogenides in the form of nanostructures to protect the surface from undesired passivation (however, in some cases, such passivation could be beneficial). Taking that into account, the typical procedure of synthesis of chalcogenide nanomaterials is quite similar to the one presented for oxide nanostructures but with the difference that now, a source of chalcogen (instead of oxygen) is used, and there is a significant accent on the stabilization of the product.

Chalcogenides reflect a group of elements; thus, the number of chalcogenide nanostructures will be much greater than in the case of oxides. In this review, chalcogenides are divided into two groups: binary compounds (two elements—one metal and one chalcogen) and multi-elemental compounds (more than one metal and one or more chalcogen).

### 5.1. Sonochemical Synthesis of Binary Chalcogenide Nanostructures and Their Applications

Cadmium sulfide (CdS) and selenide (CdSe) nanoparticles were sonochemically synthesized by Kristl et al. [91]. NPs were prepared in aqueous solutions of water-soluble cadmium salt (CdSO_4_), and the source of chalcogen was elemental sulfur or selenium. The prepared CdS NPs were spherical in form and cubic in terms of crystal structure; their average size was 4.5 nm [91]. The preparation of CdSe NPs was achieved in two ways, both sonochemical, which allowed for their comparison. The first way used NaOH to dissolute Se, while the second approach used Na_2_SO_3_ for that purpose [91]. Both procedures resulted in CdSe NPs of regular crystal structure but of spherical morphology and size in the range of 8–10 nm as revealed by TEM observations [91].

Another sonochemical approach for the synthesis of CdS NPs was attempted by Phuruangrat et al., who prepared nanorods of CdS with a hexagonal crystal structure [92]. This synthesis was conducted using a non-aqueous solvent, C_4_H_9_NH_2_, in which CdCl_2_ was dissolved and (NH_4_)_2_S was used as a source of sulfur [92]. The sonication was relatively long (5 h), and the final CdS NPs were obtained after the subsequent hydrothermal step [92]. The size of the prepared CdS nanorods was not exactly determined; however, NPs exhibited increased photoluminescence (blue-shifted in comparison to bulk CdS) after the hydrothermal step [92].

Esmaeili-Zare et al. investigated deeply the effect of different parameters of sonochemical synthesis on the properties of produced HgSe [93]. NPs were obtained in the reactions of mercury salt with SeCl_4_ in the presence of triethanolamine (TEA), which served as a complexing agent stabilizing the synthesized nanoparticles [93]. Two reducing agents were applied: potassium borohydride, KBH_4_, hydrazine hydrate, N_2_H_4_ × H_2_O, and metallic Zn [93]. The used precursor of mercury was one from the following list: HgCl_2_, HgBr_2_, Hg(NO_3_)_2_, and Hg(CH_3_COO)_2_. Synthesized HgSe NPs were rather spherical, while their size changed with sonication time, temperature, and variation in the reducing agent and mercury precursor [93]. Another study by the same authors investigated the influence of capping agents on sonochemically prepared HgSe NPs [94]. The procedure was similar to the previous one; however, a list of different capping agents was used: triethanolamine (TEA), cetyltrimethyl ammonium bromide (CTAB), and sodium dodecyl benzene sulfonate (SDBS) [94]. The main result of this study was that the utilization of SDBS as a capping agent led to the significant agglomeration of prepared HgSe NPs which inadvertently depended on sonication time—the higher the sonication time, the lower the agglomeration degree [94,95]. Interestingly, the usage of CTAB as a capping agent prevented the formation of HgSe and led to Hg_2_Br_2_ as a product, highlighting the importance of the careful selection of the composition of the reaction mixture in the sonochemical synthesis [94]. Kristl et al. demonstrated the sonochemical synthesis of the whole family of nanocrystalline HgS, HgSe, and HgTe in a simple process using water as a solvent [96]. The interesting influence of solvent on the shape of sonochemically synthesized HgSe nanorods was observed by Ding and cooperators [97]. In their study, the solvent was varied among ethylene glycol, diethylene glycol, and polyethylene glycol 200 [97]. The solvent acted as a reducing agent at the same time, and the resulting nanorods of HgSe were in the non-trivial form of tapers [97]. At the end of the plot of mercury-derived, sonochemically obtained nanostructures, it is worth mentioning that a non-trivial mercury precursor was used—Hg(salophen) (N-N′-bis(salicylaldehyde)-1,2-phenylenediimino mercury)—which allowed for the sonochemical synthesis of HgSe NPs of size mostly in the range of 20–40 nm [98].

**Figure 20:**
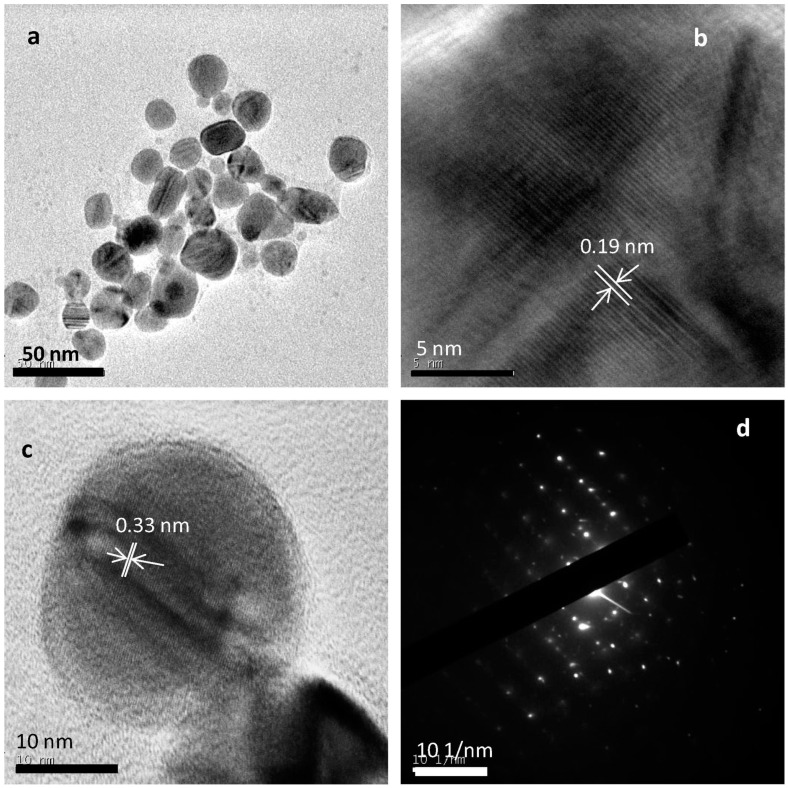
Sample TEM images of CuS NPs obtained in Singh et al. study [101]. NPs were obtained at sonication time of 60 min at 45 °C and air dried (without annealing). (**a**) TEM image showing the morphology, (**b**) TEM image showing the lattice spacing of 0.19 nm—monoclinic Cu_1.8_S, (**c**) TEM image showing the lattice spacing of 0.33 nm—Cu_2_S and (**d**) SAED pattern of synthesized particle. Reproduced under the Creative Commons license from [101].

Among the copper-derived chalcogenides that were obtained sonochemically, CuS and Cu_3_Se_2_ may be mentioned [99,100,101]. Phuruangrat et al. have obtained CuS hexanoplates and NPs starting from copper acetate (Cu(CH_3_COO)_2_) and elemental sulfur (S) dissolved by sodium hydroxide (NaOH) [99]. The reagents were mixed with ethylene glycol, and the resulting mixture was sonicated in an ultrasonic bath generating an ultrasound frequency of 35 kHz for 5 h [99]. In the same study, a concurrent approach was made, using Na_2_S as a source of sulfur instead of S and NaOH, while keeping the rest of the parameters the same [99]. This study is an interesting example of control of the morphology of resulting NPs, as synthesis using S and NaOH yielded CuS in the form of hexananoplates, and synthesis starting from Na_2_S yielded smaller cuboid nanoparticles [99]. On the other hand, Singh et al. attempted to sonochemically synthesize copper sulfide NPs in a single step using thiourea as a source of sulfur (copper acetate was a source of Cu) [101]. The obtained particles were a mixture of chalcocite (Cu_2_S), covellite (CuS), and djurleite (Cu_31_S_16_); thus, the optical bandgap of products of syntheses was in the wide range of 1.3–2.1 eV depending on the synthetic conditions [101]. The shape of the obtained NPs varied from spherical, through plate-like, to nanorods (see, for example, Figure 20) [101]. Zeinodin et al. successfully attempted to produce In-doped CuS NPs in the sonochemical synthesis [102]. They obtained In-doped nanocrystallites of c.a. 9 nm in size and utilized them as photocatalysts in the photodegradation of Methyl Orange, as doping by In slightly modified the value of optical energy bandgap of CuS NPs [102]. In-doped CuS NPs showed better degradation efficiency than the undoped ones; however, the authors linked this improvement to the change in the morphology of NPs (caused by doping with In) and the consequent increase in the surface-to-volume ratio [102].

Cu_3_Se_2_ NPs were obtained similarly to CuS NPs [100]. Copper acetate was used as a Cu source, while Na_2_Se was used as a Se source [100]. However, despite these similarities, the stoichiometry is quite different. Optimized parameters of sonochemical syntheses were as follows: sonication time 30 min, ultrasound power 200 W, and molar ratio of precursors Cu:Se = 1:2 [100]. The resulting Cu_3_Se_2_ NPs were spherical and agglomerated [100].

A family of silver chalcogenides was sonochemically synthesized by Kristl et al. [103]. The source of silver was AgCH_3_COO or AgNO_3_, and elemental chalcogens (S, Se, Te) were utilized while using ethylenediamine as a solvent [103]. The authors obtained monodisperse Ag_2_Se NPs, and Ag_2_S NPs were agglomerated while silver tellurides (with varied stoichiometry) formed clusters [93]. The prepared nanoparticles varied in size from 15 nm up to 41 nm [103]. Interestingly, Ag_2_S and Ag_2_Se were typical stoichiometries, while for the silver–tellurium system, not only Ag_2_Te was obtained, but in some cases, the formation of Ag_7_Te_4_ was also observed [103]. Jafari et al. applied a different approach for the sonochemical synthesis of Ag_2_Se NPs [104]. They used SeCl_4_ as a source of selenium, and additionally thiourea (CH_4_N_2_S) and hydrazine hydrate (N_2_H_4_ × H_2_O) as reducing agents [104]. The size of the produced Ag_2_Se NPs may be controlled by changing the molar ratio of precursors Ag:Se in the range of 4–100 nm [104]. Syntheses were conducted in aqueous solutions [104].

PbS NPs were obtained starting from lead acetate Pb(CH_3_COO)_2_ and thioacetamide CH_3_CSNH_2_ dissolved in ethylene glycol [105]. The mixture was sonicated in an ultrasonic bath generating an ultrasound of 35 kHz frequency [105]. On the other hand, PbSe NPs were sonochemically synthesized starting from [bis(salicylate) lead(II)] ([Pb(Hsal)_2_]) in aqueous solution [106]. Synthesized PbSe NPs had an average diameter of 12 nm, and ultrasonic radiation was found to be crucial for obtaining pure cubic PbSe nanostructures [106]. Under these conditions, the varied morphology of PbSe NPs was observed—from spherical-like, through nanosheets, to nanorods [106]. Nano-PbSe was also obtained in the form of a core–shell structure with PbSO_4_ (PbSe@PbSO_4_), this time utilizing [Pb(HAP)_2_] ([bis(2-hydroxyacetophenato)lead(II)]) as a precursor of Pb [107]. The procedure was similar to the case of standalone PbSe NPs; however, SDS (stabilizing agent) was present in the reaction mixture, and the calcination of prepared PbSe NPs led to the formation of a hybrid PbSe@PbSO_4_ nanostructure [107]. Both standalone PbSe NPs as well as the core–shell PbSe@PbSO_4_ were successfully utilized as photocatalysts in the photodegradation of dye Methylene Blue under UV light, showing a high yield of degradation of 45 and 98%, respectively, after 120 min of illumination [107].

Sonochemical synthesis allowed for the production of the following NPs of nickel and cobalt sulfides: NiS, Ni_3_S_4_, CoS_1.097_, and Co_9_S_8_ [108]. The average crystallite sizes in these products were in the range of 7–30 nm, while optical energy bandgaps were in the range of 3.3–3.8 eV [108]. Karekar et al. compared the conventional (just stirring) and sonochemical synthesis of molybdenum sulfide NPs (MoS_2_) and found that the ultrasound-assisted method yielded much more crystalline product than the conventional method, and it also had better UV-light absorption properties [109]. Additionally, SEM observations revealed that sonochemically obtained MoS_2_ NPs had plate-like shapes, while those obtained conventionally were rather agglomerated and random [109].

**Figure 21:**
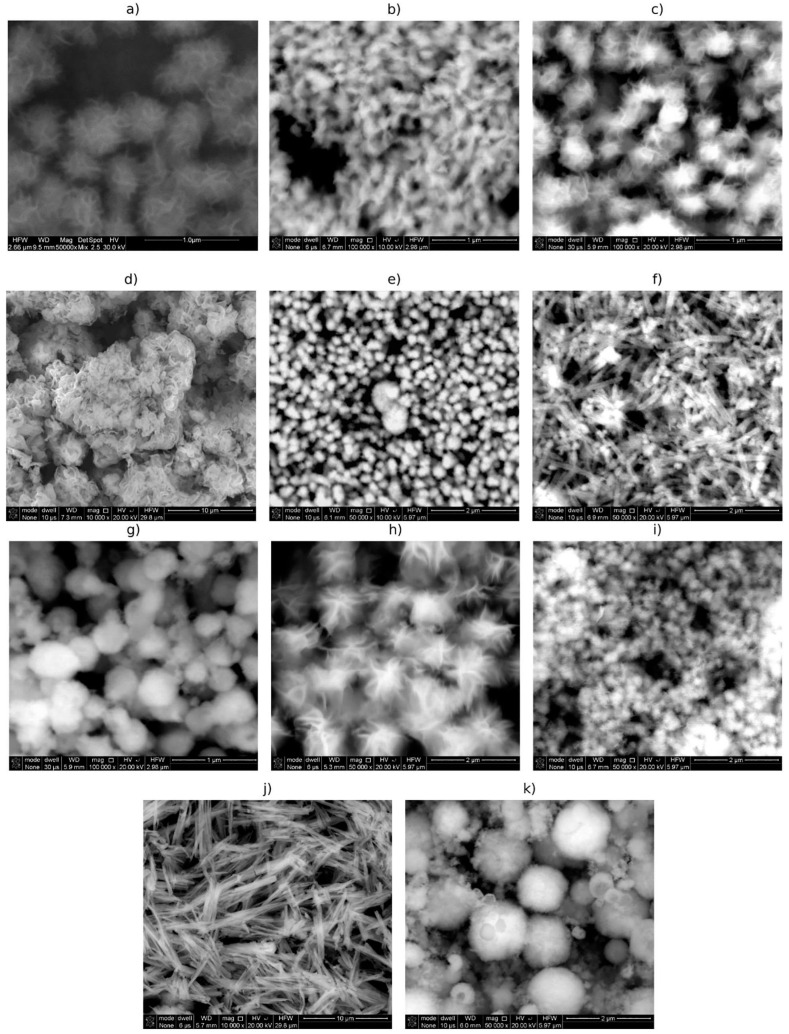
SEM images illustrating many morphologies of sonochemically synthesized powders of tin sulfides. Experimental conditions (tin source, solvent, time, TAA:tin source ratio, product): (**a**) SnCl_2_, ethanol, 160 min, 2.5, SnS_2_; (**b**) SnCl_2_, ethanol, 100 min, 2.5, SnS_2_; (**c**) SnCl_2_, ethanol, 230 min, 1, SnS_2_; (**d**) SnCl_4_, ethanol, 100 min, 2.5, SnS_2_; (**e**) SnCl_2_, ethanol, 100 min, 3.5, SnS_2_; (**f**) SnCl_4_, ethanol, 100 min, 3.5, SnS_2_; (**g**) SnCl_2_, ethylenediamine, 100 min, 3.5, SnS; (**h**) SnCl_4_, ethanol, 160 min, 2.5, SnS_2_; (**i**) SnCl_2_, ethanol, 160 min, 3.5, SnS_2_; (**j**) SnCl_2_, ethylenediamine, 160 min, 2.5, SnS_2_; (**k**) SnCl_2_, ethylenediamine, 160 min, 3.5, SnS. Reproduced under the Creative Commons license from [113].

The application of ionic liquids as solvents in the sonochemical synthesis of chalcogenide nanostructures was demonstrated in the examples of Sb_2_S_3_ and SnS [110,111]. Sb_2_S_3_ NPs were obtained from Sb_2_Cl_3_, thioacetamide, ethanol and ionic liquid 1-butyl-3-methylimidazolium tetrafluoroborate ([BMIM][BF_4_]) [110]. The addition of ionic liquids soundly influences the morphology of resulting products. In the case of Sb_2_S_3_, thanks to the usage of [BMIM][BF_4_] as a solvent, crystalline nanorods are prepared while without ionic liquid, Sb_2_S_3_ NPs were of round morphology [110]. SnS NPs were sonochemically synthesized from an analogical reaction mixture as Sb_2_S_3_ (SnCl_2_, thioacetamide, ethanol, same ionic liquid [BMIM][BF_4_]) [110,111]. Garcia-Gomez et al. showed that also in this case, the morphology of the produced SnS NPs is strongly influenced by the composition of the reaction mixture (i.e., the ratio of ionic liquid to ethanol) and by the ratio of precursors of S and Sn [111]. Varying these parameters, it was possible to produce irregular polygons, regular polyhedra, platelets, grape-like agglomerates, and mesoporous-like nanostructures [111]. Matyszczak et al. also studied the sonochemical synthesis of tin sulfides (SnS and SnS_2_), showing that by varying the solvent, it is possible to obtain nano- and micropowders with particle sizes ranging from single nanometers (quantum dots synthesized in water) through tens of nanometers (SnS nanoparticles obtained in ethylenediamine) to hundreds of nanometers (SnS_2_ sub-microparticles obtained in ethanol starting from SnCl_2_) (see Figure 21) [5,112,113]. Sonochemically synthesized nanoparticles of tin sulfides found applications in the sono- and photocatalytic removal of Metanil Yellow and also as coatings for electrodes in the electro-Fenton process [5,112,113,114].

**Figure 22:**
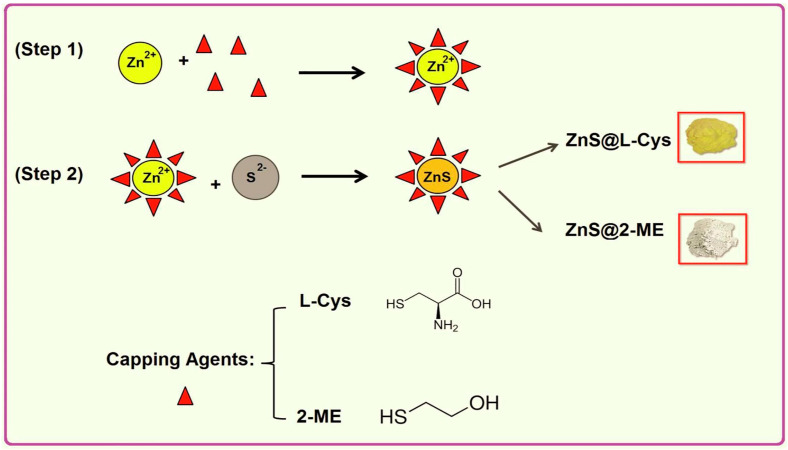
The capping agent effect in synthesis of ZnS QDs. Reproduced under the Creative Commons license from ref. [6].

The capping agent effect was discussed in the synthesis of ZnS QDs [6]. The research checked two capping agents: 2-mercaptoethanol and l-cysteine. They are added (only one-for-one synthesis) to a solution of zinc ions to cover it and form a stable complex with a protective layer on the NPs surface that prevents their further growth (Figure 22) [6].

### 5.2. Sonochemical Synthesis of Multi-Elemental Chalcogenide Nanostructures and Their Applications

Nanostructures of AgInS_2_ were sonochemically obtained by Hosseinpour-Mashkani et al. utilizing a novel precursor of Ag, silver(I) salicylate, [Ag(HSal)] [115]. The source of In was InCl_3_, and the source of sulfur typically was a thioacetamide; propylene glycol was utilized as a solvent [115]. The experiments proved that while using [Ag(HSal)] as a silver precursor, the addition of CTAB to the reaction mixture did not influence the size of the prepared AgInS_2_ NPs [115]. The opposite observation was made while using AgNO_3_ as a silver precursor [115]. The prepared AgInS_2_ NPs were used in FTO/AgInS_2_/CdS/Pt-FTO solar cells, however, reaching an efficiency of less than 0.5% [115].

CuInS_2_ NPs were obtained quite similarly to the AgInS_2_ NPs but using copper and indium nitrates as respective precursors [116]. Nanoparticles of CuInS_2_ exhibited a flexible value of direct optical energy bandgap in the range of 1.33–1.57 eV, depending on the relative concentrations of Cu and In precursors in the reaction mixture [116]. Such values of optical energy bandgap are good for applications in solar cells (i.e., they lie in the so-called Schockley–Queisser limit)—the authors constructed a glass/Mo/CuInS_2_/CdS/i-ZnO/ZnO:Al solar device which exhibited an energy conversion efficiency of c.a. 1% [116].

Another material widely investigated in the context of applications in photovoltaics—kesterite, CZTS—was also achieved in sonochemical synthesis [117]. Paraye et al. used common, water-soluble salts as precursors of Cu, Sn, and Zn together with citric acid as a complexing agent [117]. Sodium thiosulfate (Na_2_S_2_O_3_) was applied as a sulfur source. The reaction mixture was sonicated in an ultrasonic bath at two different frequencies of ultrasound: 25 kHz and 45 kHz [117]. CZTS NPs synthesized under 45 kHz showed properties more relevant for applications in solar cells [117].

## 6. Sonochemical Synthesis of Biologically Active and Bio-Derived Nanostructures and Their Applications

Nanospheres of DNA were produced utilizing five distinct types of DNA: genome DNA originating from lettuce leaves and parasites, plasmid DNA, linear DNA, single-stranded DNA, and double-stranded DNA. A portion of certain DNA was first extracted to an aqueous phase which was subsequently sonicated after the addition of an organic phase consisting of dodecane or soy oil, which was taken in a defined amount [7]. An ultrasonic probe generating high-frequency ultrasound was used, and the temperature of the process, which lasted 3 min, was 22 °C. Synthesized DNA nanospheres were then coated with albumin protein from the bovine serum. The nanospheres of DNA were prepared both in the atmosphere of air as well as in the atmosphere of argon. It was observed that a higher yield of nanospheres was achieved under argon atmosphere [7]. The stability of DNA nanospheres was tested under different conditions including high temperature, pressure, and the presence of DNA hydrolytic enzymes [7]. It turned out that the most stable were nanospheres obtained starting from dsDNA in comparison to those obtained starting from ssDNA, which may be caused by the different stabilizing interactions present in both types of DNA. Double-stranded DNA is stabilized by strong covalent bonds while the single-stranded DNA is stabilized by much weaker electrostatic forces. The relatively good stability of DNA nanospheres was utilized as a base for the application of sonochemically derived DNA nanospheres in medicine in biology. For example, in genetics, they allow the incorporation of foreign genes into bacterial cells for the purpose of expression of new features [7]. DNA nanospheres were also able to penetrate human cancer cells [7].

Despite the positives of DNA, RNA is more versatile and flexible in the context of the formation of nanostructures [8]. The manipulation of RNA, however, is relatively hard due to their high lability–RNA samples may degrade even under cold and enzyme-free conditions [8]. The increase in stability of RNA is an important issue for the development of advanced therapeutics. Shimanovich et al. have shown that ultrasonic waves are capable of converting RNA molecules to RNA nanospheres which are stable at room temperature for at least one month [8]. In the same study, these nanospheres of RNA were also successfully incorporated into mammalian cancer cells [8]. Interestingly, the RNA nanospheres were able to reversibly refold to original molecules under denaturing conditions.

Other examples of biologically active nanomaterials are bioceramic hydroxyapatite (HA) and fluorapatite (FA) in the form of nanoparticles which may be widely utilized in tissue engineering and biomedicine [118]. As these compounds consist of calcium phosphate, they may be produced starting from calcium nitrate, ammonium hydrophosphate, ammonium fluoride, ethanol, and ammonia as additional reagents [118]. To synthesize hydroxyapatite, in the presence of an ultrasound probe, a solution of calcium nitrate in ethanol was added to an aqueous solution of ammonium hydrophosphate [118]. The procedure to synthesize fluorapatite was similar but with the addition of ammonium fluoride. The course of the reaction was evidenced due to the change in mixture color. In the end, a few drops of aqueous ammonia were added to the reaction mixture, and the resulted precipitates were collected, washed with ethanol, and subsequently calcined [118]. Due to the presence of fluorides in the structure of FA, the FA NPs were more crystalline than the HA NPs [118]. Both nanopowders were of high purity; however, they tended to form agglomerates of NPs. FA and HA may be used for the preparation of bio-nano-ceramics utilized as substitutes for the building of bones for both orthopedy and stomatology.

**Figure 23:**
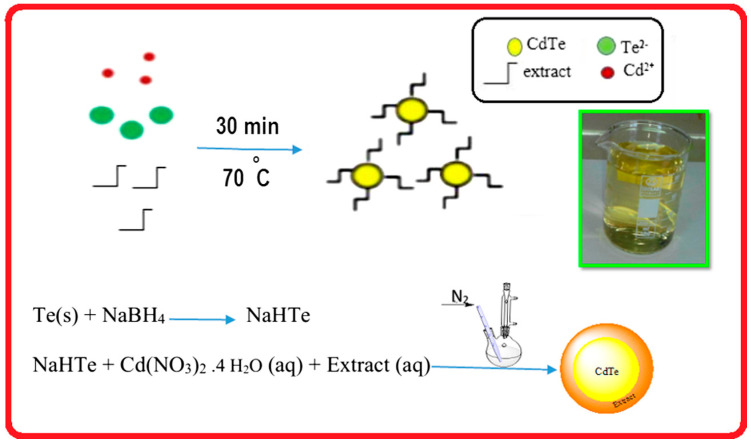
Scheme of sonochemically assisted synthesis of CdTe QDs with the utilization of extract from seeds and leaves of Ficus johannis. Reproduced with consent from [119].

Growing interest is also observed in the field of utilization of natural extracts (from plants) as additives to reaction mixtures in sonochemical synthesis. Such an approach helps reduce the usage of toxic and harmful chemical compounds. Extracts from plants contain a variety of organic compounds that may act as reducing, stabilizing, and capping agents in the sonochemical synthesis [119]. For example, CdTe QDs were synthesized with the usage of aqueous extracts from powdered fruits of Ficus johannis [119]. This extract was obtained by mixing powdered fruits with distilled water and sonicating them in an ultrasonic bath [119]. Subsequently, the extract was joined with an aqueous solution of cadmium nitrate under the N_2_ atmosphere as well as with tellurium and NaBH_4_. The prepared CdTe QDs exhibited antibacterial and antifungicidal activities [119]. Figure 23 depicts a schematic representation of this synthesis.

The next example of biologically active nanomaterials is materials with combined magnetic and plasmonic properties. Such hybrid nanomaterials have a magnetic part (consisting of, e.g., Fe_2_O_3_) and a plasmonic part (consisting of noble metal, like Au) [120]. Nanomaterials with such dual properties may be used for magnetothermal and photothermal therapy against cancer. Ruiz-Baltazar additionally showed that hybrid Au/Fe_2_O_3_ nanomaterial may be also utilized as a sonocatalyst for the removal of Methyl Orange and possibly other organic compounds [120]. In the synthesis of Au/Fe_2_O_3_ nanocomposite, a plant extract was used (from Piper auritum)—a mixture of leaves and water was heated and the extract was joined with an aqueous solution of HAuCl_4_, and subsequently such mixture was subjected to ultrasound [120]. At the end, aqueous solutions of FeCl_3_ and FeCl_2_ were added under sonication conditions. The resulting magnetic NPs were washed with isopropanol [120].

Nanomaterials based on graphene may be used not only in batteries, fuel cells, or solar cells but may also be applied as electrochemical nanocatalysts. For example, a nanocomposite made of Fe compound and graphene was used for the quantitative detection of uric acid, which is an important marker of cancer diseases [121]. On the other hand, in biomedical engineering, Pt NPs originating from plant extracts were used as an alternative to iodine as a contrast in computer tomography [15]. In comparison with iodine compounds, Pt NPs have many advantages, including the high absorption of X-rays as well as the ease and control of synthesis [15]. Supermagnetic Fe NPs were additionally utilized in medicine as cell markers and drug carriers [3]. Due to the relatively low toxicity of substrates and products of their synthesis, as well as to mild temperature conditions, syntheses of such NPs may likely be adopted on a large, industrial scale, which would revolutionize the medical market [3].

## 7. Summary

Nowadays, the sonochemical technique is a simple, fast, and cost-effective method for the synthesis of nanomaterials with different shapes and sizes. Ultrasound cavitation opens the way for nanostructures with unique properties, but at the same time, numerous factors must be taken into consideration when planning the synthesis. The reaction time, temperature, ultrasound power, precipitating agent, addition of capping agent, and doping influence the properties of nanoparticles; therefore it is possible to develop a synthesis of nanomaterials for use in various fields. In the last decade, there has been a strong trend toward the production of metals, carbon, oxides, and chalcogenides in the form of nanomaterials and nanocomposites where a heterojunction effect enables the creation of unique properties. A lot of materials were achieved on a laboratory scale and proven to be effective, but large-scale production is still in the early stages. In order to adapt the sonochemical method to an industrial scale, sonochemical reactors and sources of ultrasound should be developed to make an effective combination of chemical reactions and physical effects of ultrasonic cavitation. Due to the possibility of reduction in toxic wastes and reagents in the sonochemical processes, such methods are likely to be implemented in industry in the future.

## Figures and Tables

**Table 1:** The overview of basic parameters of selected sonochemical syntheses of metal NPs synthesized in the solution.

No.	Obtained NPs	Metal Precursor	Solvent	Other Reagents	Ultrasound	Ref.
1.	Irregular, icosahedral, nanorods–gold NPs	HAuCl_4_	Water	Isopropanol, SDS (stabilizing agent)	463 kHz	[20]
2.	Gold nanosheets	HAuCl_4_	Water	Tryptophan (reducing and capping agent)	490 kHz	[17]
3.	Gold NPs (12–16 nm)	HAuCl_4_	Water	Trisodium citrate	20 kHz	[4]
4.	Spherical gold NPs	HAuCl_4_	Water	Purged gases (H_2_, N_2_, Ar, O_2_)	950 kHz	[21]
5.	Platinum NPs (3.5 nm)	K_2_PtCl_4_	Water	Plant extract (reducing agent and stabilizer)	20 kHz	[18]
6.	Platinum NPs (2.2 nm, 2.3 nm)	PtCl_4_	Water–ethanol mixture	NaBH_4_ (reducing agent), purged Ar	20 kHz, 408 kHz	[19]
7.	Silver NPs (4.21 nm)	AgNO_3_	Water	κ-carrageenan (stabilizer)	50/100 Hz	[22]
8.	Silver NPs	AgNO_3_	Water	NaBH_4_ (reduction), purged N_2_	NA	[23]
9.	Cu NPs (167–520 nm)	Cu(CH_3_COO)_2_	Ethylene glycol	C_6_H_8_O_6_ (ascorbic acid, reducing agent)	40 kHz	[24]

**Table 2:** The overview of basic parameters of selected sonochemical syntheses of metal NPs on surfaces or supports.

No.	Obtained Structure	Surface/Support	Solvent and Other Reagents	Ultrasound	Ref.
1.	Gold NCs	Silica-Coated CdSe-Dot/CdS-Rod nanocrystals	Polyethylene glycol	35 kHz	[28]
2.	Copper and nickel NPs	Ceramic (Al_2_O_3_ and TiO_2_)	Water, L-ascorbic acid (reducing agent), CTAB (dispersant)	20 kHz	[25]
3.	Cu NPs	TiO_2_	L-ascorbic and CTAB	various	[29]
	Ag NPs		NaBH_4_ and PVP40		
	Mn NPs		HNO_3_, EtOH, Ti(IV)-buOX		
4.	Mo and Pd nanomaterials	Activated carbon and multiwall carbon nanotubes	Hexadecane, ethanol, ethylene glycol, polyethylene glycol, and ionic liquids as solvents	20 kHz	[30]
5.	Ag_3_PO_4_ QDs	Graphite oxide	Water, ethanol, sodium oleate, Na_2_HPO_4_	20 kHz	[27]
6.	Silver QDs	Exfoliated graphitic carbon nitride (g-C_3_N_4_)	Water, extract from ginseng root	40 kHz	[26]

**Table 3:** Summary of areas of application for sonochemically obtained metal nanostructures.

No.	Application	Example	Ref.
1.	Catalysis	Gold nanosheets in the reduction of 4-nitrophenol	[17]
		Au@Pd core–shell NPs for reduction of 4-nitrophenol	[31]
		AlNi alloys in hydrogen evolution reaction	[35]
1.1	Photocatalysis	Removal of organic compounds in water by Ag/Cu/Mn NPs on TiO_2_	[29]
		Hydrogen production by Ag QDs on g-C_3_N_4_	[26]
1.2	Electrocatalysis	Pd_3_Mo NPs on carbon substrates for oxygen reduction reaction for fuel cell application	[30]
		Pt–Carbon black nanocomposite	[37]
2.	Metal−semiconductor hybrid systems	Gold nanocrystals on the silica-coated CdSe-dot/CdS-rod core/shell NCs	[28]
3.	Antimicrobial activity	Ag QDs on g-C_3_N_4_	[26]

**Table 4:** Summary of sonochemically obtained CQDs and metal-doped CQDs properties [45].

Property	CDs	Ga@CDs	Sn@CDs	Zn@CDs	Ag@CDs	Au@CDs
QY (%)	16	1.8	19	10	2	2
Size (nm)	5	6	6	7	8	7
Doping level (ppm)	-	340	410	230	440	440

**Table 5:** Summary of synthesis methods and potential applications of different types of sonochemically synthesized carbon NPs.

Type of Nanoparticle	Method of Synthesis	Potential Uses	Ref.
Pure CQDs	Ultrasonication	Bioimaging and solar cells	[39,45]
TiO_2_/GQD systems	Oxidation with acid combined with ultrasonication and calcination	Photocatalysis, solar cells	[54]
Pd-GO nanocomposites	Sonication in water bath	Hydrogen storage	[44]
g-C_3_N_4_/CQDs nanocomposite	Ultrasonic exfoliation	H_2_O_2_ production	[46]
N@CQDs/SnO_2_ nanocomposite	Hydrothermal and sonochemical	Voltammetric sensor for riboflavin detection	[43]
CQDs@Ln-MOFs nanocomposite	Hydrothermal and sonochemical	White light-emitting diodes with high color rendering	[56]
N@Graphene sheets	Ultrasonication	Oxygen reduction in alkaline media	[47]
Sn@CQDs/Sn nanocomposite	Ultrasonication	Anode for Li-ion batteries	[53]
N@CQDs	Ultrasonication	Sensitive detection of Fe^2+^ ions, nanoprobe for cancer cell detection (bioimaging)	[40,42]
Ga@CQDs	Ultrasonication	Singlet oxygen production	[52]
S@Carbon nitride ribbons	Hydrothermal and sonochemical	Photocatalytic reduction of CO_2_	[51]
ZnS NPs@rGO nanocomposite	Ultrasonication (GO synthesized by modified Hummers method)	Electrode for detection of hydroxychloroquine and daclatasvir in human plasma and urine	[57]
M@CQDs(M = Ag, Au, Ga, Sn, Zn)	Ultrasonication	Cell labeling	[45]

**Table 6:** Summary of procedures of syntheses and applications of sonochemically synthesized oxide nanostructures.

Nanomaterial	Synthesis Description	Additional Information	Application	Ref.
Cu_2_O	Cu(CH_3_COO)_2_ in glycerol US (20 kHz, 750 W, with 2 s pulse rate) for 1 h		catalyst	[65]
Fe_2_O_3_/ZrO_2_	FeSO_4_ + ZrOCl_2_ in water + ethanol + propanol US for 50 min/calcination 600 °C, 2 h/HAuCl_4,_ pH 10, US for 30 min	Different Fe/Zr molar ratios (0.5, 1, and 2) were used and different amounts of Au (2.5 and 5% wt.) were added	catalyst	[66]
CeO_2_	Ce(CH_3_COO)_3_ + NaOH + ionic liquid in water stirred for 30 min/US for 12 h	Different cerium sources (cerium acetate, cerium nitrate and, cerium chloride), precipitating agents (NaOH and NH_4_OH), and ionic liquids ([Edimim][Tf_2_N], [Pyrr_14_][Tf_2_N], [C_4_Py][Tf_2_N], and [N_1112_OH][Tf_2_N]) were used	catalyst	[67]
Ho_2_Cu_2_O_5_/Ho_2_O_3_	Cu(NO_3_)_2_ + Ho(NO_3_)_3_ + TEPA in water US (45 W) then stirred at 80 °C for 20 min/calcination at 700 °C and 1000 °C	Different sonication times of 10, 20, 30 min. With a sonication time of 10 min and different pulse rates of 0.1 and 0.5 s	photocatalyst	[68]
Ce-doped TiO_2_	Ce(NO_3_)_3_ + P-123 (a surfactant) + Ti(OCH(CH_3_)_2_)_4_ + NaOH in water US (20 kHz) for 1 h/calcination at 500 °C for 1 h	Different Ce ratios were used (0.5%, 0.75%, 1.0%, 1.5%, and 2.0%). The best catalytic performance was achieved with 0.75% wt. Ce.	photocatalyst	[69]
Graphene-Ce-TiO_2_ and Graphene-Fe-TiO_2_	For graphene-Ce-TiO_2_ synthesis Ti(OCH(CH_3_)_2_)_4_ + GO in 2-propanol US for 30 min/NaOH and Ce(NO_3_)_3_ separate water solutions were added during sonication for 30 min/calcination at 300 °C for 3 h.	For graphene–Fe–TiO_2_ synthesis, ferric nitrate was taken instead of cerium nitrate. Graphene–Fe-TiO_2_ showed the best kinetic rate	photocatalyst	[70]
Ho-doped ZnO	Ho(NO_3_)_3_ + ZnCl_2_ + NaOH in water US for 3 h (36 kHz)	Different amount of Ho was added (2, 4, and 6 mol% Ho). 4% Ho-doped ZnO is the most efficient catalyst	photocatalyst	[71]
MnFe_2_O_4_	MnSO_4_ + FeSO_4_ + NaOH in water US for 2 h/calcination at 650 ± 10 °C for 2 h	The degradation percentage of Methylene Blue was 96% while Drimaren Yellow was only 7%	photocatalyst and sensor	[72]
Iron oxide	FeCl_3_ + FeSO_4_ in water US and mixed in 60–70 °C/NaOH added and US for 1 h		toxic metal remediation	[77]
Al_2_O_3_	Al(NO_3_)_3_ + NH_4_OH in water US for 1 h/calcination at 500 °C for 4 h/received Al_2_O_3_ sonicated with APTES in isopropanol/resulted product in DCM + indole in acetonitrile US for 1 h	Instead of indole, indole-2 carboxylic acid and 2-methyl indole were used. Sonochemically obtained aluminum hybrids were compared to synthesized without sonication ones, and sonicated products showed better adsorption.	toxic metal remediation	[78]
NiMnO@pr-GO	NiCl_2_ + MnCl_2_ + NaOH in water US (37 kHz, 150 W) for 2 h/calcination at 550 °C for 3 h/NiMnO + GO in ethanol US for 15 min		sensor	[79]
GOS decorated with TiO_2_	Ti(OCH(CH_3_)_2_)_4_ in ethylene glycol US (50 kHz and 100 W/cm^2^) at ambient air for 30 min/calcined at 600 °C for 3 h/received TiO_2_ + GOS US for 30 min		sensor	[80]
MnFe_2_O_4_/C_3_N_4_	FeCl_3_ + MnSO_4_ in water US (30 kHz/70 W) for 30 min/calcination at 450 °C for 2 h/MnFe_2_O_4_ + C_3_N_4_ in water US for 30 min		sensor	[81]
Co_3_O_4_/NRGO	Co(CH_3_COO)_2_ + NRGO in water US for 30 min (480 W/cm^2^, 24 kHz) then NH_4_OH was added over 15 min	In another synthesis, RGO was used instead of NRGO to receive Co_3_O_4_/RGO	capacitor	[82]
NiO	NiCl_2_ + NaOH in water US for 1 h/calcination at 250, 450 and 650 °C		capacitor	[83]
Co_3_O_4_	Co(CH_3_COO)_2_ + NaOH in water US for 1 h/calcination at 300 °C for 3 h		capacitor	[84]
CeO_2_	Ce(NO_3_)_3_ stirred with mentha extract in water/US (100 W) for 12 min with dropwise addition of NH_4_OH/calcination at 500 °C for 2 h		cancer therapy	[87]
CuO and ZnO-coated modified PE	Cu(OAc)_2_ in water-ethanol (1:9 by volume) US in the presence of modified PE for 30 min/heating to 60 °C dropwise addition of NH_4_OH	In the same synthesis, zinc acetate was used instead of copper to obtain ZnO coating	hydrophobic and antibacterial	[88]
CuO and ZnO	Cu(OAc)_2_ + H_2_O_2_ in water with US Zn(OAc)_2_ + NaOH in water with US	Different US sources were used with different reaction times. Dental tip for 10 min and sonochemical horn for 60 min	antibacterial	[89]
ZnO coated cotton	Cotton immersed in water/ZnCl_2_ added/addition of NaOH to maintain pH = 9/sonication	Different reaction times were used (60 min and 120 min), different ZnCl_2_ amounts were added (10 g and 20 g), and different horn intensities were used (50% and 70%)	antimicrobial	[90]

## Abbreviations

NPs	nanoparticles
[Zn_2_(btec)(DMF)_2_]_n_	btec=1,2,4,5-benzenetetracarboxylate
DMF	N,N-dimethylformamide
FTS	Fischer–Tropsch synthesis
[Tf_2_N]^−^	bis(trifluoromethanesulfonylamide)
[C_4_mim]^+^	1-butyl-3-methylimidazolium
[Edimim]^+^	1-ethyl-2,3-dimethylimidazolium
[C_4_Py]^+^	butyl-pyridinium
[Pyrr_14_]^+^	1-butyl-1-methyl-pyrrolidinium
[N_1112_OH]^+^	2-hydroxyethyl-trimethylammonium
TEPA	tetraethylenepentamine
P-123	poly(ethylene glycol)-block-poly(propylene glycol)-block-poly(ethylene glycol)
GCE	glassy carbon electrode
APTES	3-aminotriethoxypropylsilane
DCM	dichloromethane
GO	graphene oxide
RGO	reduced graphene oxide
GOS	graphene oxide sheets
NRGO	nitrogen-doped reduced graphene oxide
GCN	graphitic carbon nitride
PVDF	polyvinylidene fluoride
NMP	N-methylpyrrolidone
QDs	quantum dots
dsDNA	double-stranded DNA
ssDNA	single-stranded DNA
HA	hydroxyapatite
FA	fluoroapatite
GNPs	gold nanoparticles
PSCN	porous S-doped carbon nitride
MOF	metal–organic framework
UFBs	ultrafine bubbles
NA	not available
g-C_3_N_4_	graphite–carbon nitride
WLEDs	white light-emitting diodes
CTAB	cetyltrimethyl ammonium bromide
PVP40	polyvinylpyrrolidone (of average molecular weight 40,000)
SDS	sodium dodecyl sulfonate
SDBS	sodium dodecyl benzene sulfonate
TEA	triethanolamine
